# Operando Soft X-ray Absorption of LaMn_1–*x*_Co_*x*_O_3_ Perovskites for CO Oxidation

**DOI:** 10.1021/acscatal.4c03259

**Published:** 2024-07-12

**Authors:** Qijun Che, Mahnaz Ghiasi, Luca Braglia, Matt L. J. Peerlings, Silvia Mauri, Piero Torelli, Petra de Jongh, Frank M. F. de Groot

**Affiliations:** † Materials Chemistry and Catalysis, Debye Institute for Nanomaterials Science, Utrecht University, Universiteitsweg 99, 3584 CG Utrecht, The Netherlands; ‡ AREA Science Park, Padriciano 99, I-34149 Trieste, Italy; § CNR-Istituto Officina dei Materiali, 34149 Trieste, Italy

**Keywords:** operando catalysis, X-ray absorption spectroscopy, charge transfer multiplet theory, spin and valence states, LaMn_1−*x*_Co_*x*_O_3_ perovskites

## Abstract

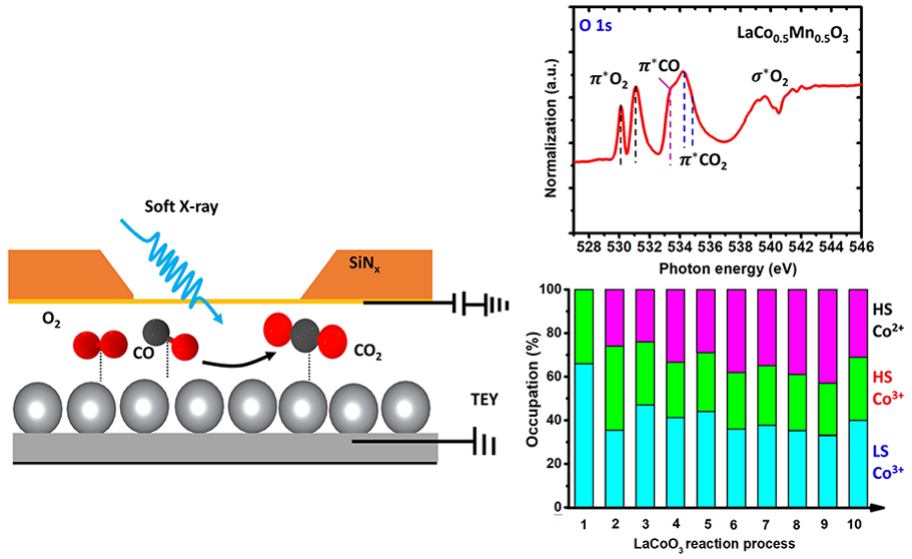

We employed operando soft X-ray absorption spectroscopy (XAS) to monitor the changes in the valence states and spin properties of LaMn_1–*x*_Co_*x*_O_3_ catalysts subjected to a mixture of CO and O_2_ at ambient pressure. Guided by simulations based on charge transfer multiplet theory, we quantitatively analyze the Mn and Co 2p XAS as well as the oxygen K-edge XAS spectra during the reaction process. The Mn sites are particularly sensitive to the catalytic reaction, displaying dynamics in their oxidation state. When Co doping is introduced (*x* ≤ 0.5), Mn oxidizes from Mn^2+^ to Mn^3+^ and Mn^4+^, while Co largely maintains a valence state of Co^2+^. In the case of LaCoO_3_, we identify high-spin and low-spin Co^3+^ species combined with Co^2+^. Our investigation underscores the importance to consider the spin and valence states of catalyst materials under operando conditions.

## 1 Introduction

The release of CO into the atmosphere has significant adverse impacts on both human health and the environment.^1^ It is imperative to develop an efficient catalyst to mitigate CO emissions from fuel vehicles. Historically, Pt-based catalysts were the first to demonstrate a high level activity of CO oxidation at temperatures below 200 °C,^2,3^ but high cost, low abundance and inferior thermal stability limit their widespread applications.^4^ Over the last decades, La-based perovskites (LaTMO_3_, TM = transition metal) have garnered attention regarding thermal catalytic reactions of CO oxidation, NO_*x*_ reduction, and hydrocarbon oxidation.^5,6^ In particular, LaCoO_3_ stands out as one of the potential catalysts for CO oxidation at moderate temperatures due to its ability to adjust its morphology, size and electronic structure.^7,8^ Lu et al.^9^ reported a mesoporous LaCoO_3_ catalyst that showed 100% CO conversion at a temperature of ∼130 °C, where the Mn incorporation could optimize the catalytic activity and thermal stability. LaMn_1–*x*_Co_*x*_O_3_ polycrystalline samples have been studied by ex-situ X-ray spectroscopy, suggesting a divalent Co^2+^ ion and a Mn^4+^–Mn^3+^ double-exchange at low concentrations of Co (15–20%), while at *x* = 0.5, the systems were found to contain a combination of Co^2+^–Mn^4+^.^10,11^

CO oxidation (2CO + O_2_ → 2CO_2_), a prototypical reaction, is important to understand the heterogeneous catalysis surface mechanism. CO_2_ cannot be formed when gaseous CO directly interacts with oxygen adsorbed at the surface, i.e., the Eley–Rideal mechanism. The reactants have to be coadsorbed onto the surface of the catalysts, resulting in the oxidation of CO, i.e., the Langmuir–Hinshelwood mechanism.^12^ The kinetic steps of CO oxidation are as follows, where (i) is an elementary step and (ii) and (iii) are combined reactions:^13^
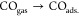
i
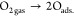
ii
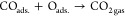
iiiBonn et al. investigated the thermal excitation and picosecond (ps) laser excitation for CO oxidation on the O-covered Ru(0001) surface in vacuum,^12^ and showed that ps-laser excitation of CO on O/Ru(0001) can give oxidation while thermal excitation cannot. Traditionally, the thermal reaction on a catalyst’s surface is driven by phonons to overcome the activation barrier, but the picosecond laser excitation reaction dynamics involve hot substrate electrons activation. Knowledge about the electronic parameters of catalysts at working conditions, such as the charge-transfer, the oxidation state, and the covalence, are important descriptors for the development of new catalysts.^6^ Recently, a number of studies on perovskites have found correlations between the electronic structure and catalytic activity in both electrocatalysis^14,15^ and thermochemical catalysis applications.^16^ Mueller et al.^17^ demonstrated that the adsorbed molecules near the Fermi level modify the electronic structure and covalency of the catalyst surface. The details of covalent bonding can be probed by oxygen K-edge X-ray absorption spectroscopy (XAS).^18^ In combination with X-ray emission spectroscopy (XES), the charge-transfer energy (Δ_ct_) can be derived. Δ_ct_ is defined as the (many-body) excitation energy between the 3d^*n*^ and 3d^*n*+1^L configurations, where L is a hole in the ligand valence band.^19^ Volcano-type plots were established in heterogeneous catalysis, where details regarding the spin state and orbital occupations in LaCoO_3_ are complex.^6,14−16^ In a LaCoO_3_ single crystal, Co^3+^ is low-spin (LS) at low temperature and gradually increases in high-spin (HS) character.^20^ The variation of the Co spin and valence state for oxidation or reduction involved in reactant interaction in catalysis depends on the reaction conditions, such as temperature and pressure.^21^ LaMnO_3_, mainly octahedrally coordinated with six oxygens, is an antiferromagnetic insulator below the Neel temperature (T_N_ ≈ 140 K), in which the Mn^3+^ ions have a half-filled e_g_ orbital and undergo a Jahn–Teller distortion.^22−24^ Mixed valence states can be formed, for example, by La and/or transition metal substitution, interface engineering, and/or nonstoichiometric oxygen. With Co incorporation, the LaMn_1–*x*_Co_*x*_O_3_ compounds can form different combinations of oxidation states of Co and Mn, including Co^2+^, Co^3+^, Mn^2+^, Mn^3+^, and Mn^4+^. Among them, the Co^3+^ site can be activated by temperature, magnetic field, pressure, and lattice strain.^25−27^

Operando characterization is vital to capture the complexity of a catalyst under working conditions. Hard X-ray XAS is commonly employed in operando catalysis studies. Transition metal K-edge XAS provides bulk information, where the near-edge structure (XANES) gives electronic structure information and the extended fine structure (EXAFS) provides geometric information. Operando soft X-ray XAS at the transition metal 2p (L_2,3_ edges) and oxygen 1s (K-edge) edge provide a different view on catalyst materials with a number of advantages:

1) Operando soft X-ray XAS detected with electron yield detection has an approximately 4 nm probing depth and as such is (near) surface-sensitive.
2) The lifetime broadening of soft X-ray XAS is ∼200 meV, which implies that the spectral features have an at least 5 times higher spectral resolution. This much improved intrinsic resolution reveals a larger detail and accuracy regarding the valence state and ground state symmetry, also due to the fact that the L_2,3_ edges directly probe the important 3d states.
3) The metal L_2,3_ edges can be combined on the same beamline with the carbon and oxygen K-edge spectra, allowing the tracking of both catalyst and reactants.

In this work, we study LaMn_1–*x*_Co_*x*_O_3_ catalysts for the CO + O_2_ reaction mechanism at ambient pressure using soft X-ray absorption spectroscopy. The TM L-edges and O K-edges have been measured by electron yield under operando conditions. Conducting operando soft XAS experiments at 1 bar gas phase reactions are not easy due to the relatively short penetration depth of soft X-rays.^28,29^ With the help of SiN_*x*_ windows, we separate the vacuum and operando environments, allowing electron yield detection by XAS.^28−32^ Using charge transfer multiplet theory simulations, we quantitatively identify the dynamics of the valence and spin states. The results are dependent on the reaction conditions and different from previous in situ studies at low pressure (0.37 kPa)^6^ and from vacuum conditions.

## 2 Experimental Methods

### Synthesis of Perovskite Co-Doped LaMnO_3_ Catalysts

The perovskite Co-doped LaMnO_3_ samples were synthesized via a sol–gel method.^33−35^ Stoichiometric amounts of metal nitrate salts (La(NO_3_)_3_·6H_2_O, Co(NO_3_)_2_·6H_2_O, and Mn(NO_3_)_2_·4H_2_O) and citric acid (∼5 times the used metal nitrate amounts) were dissolved in 250 mL of deionized water. The resultant solution was heated at 80 °C under stirring to form a gel, and at 150 °C was treated for 12 h to decompose, forming a solid. The solid was decomposed at ∼400 °C for 5 h to remove the organic components and further calcined at 900 °C for 5 h in a ceramic reactor with a ramp rate of 8 °C min^–1^ to yield the LaMnO_3_, LaCoO_3_, LaMn_0.5_Co_0.5_O_3_, LaMn_0.75_Co_0.25_O_3_, and LaMn_0.25_Co_0.75_O_3_ perovskite nanoparticles.

### Atomic Structure Characterization

Powder X-ray diffraction data were recorded at room temperature on a Bruker AXS D2 Phaser diffractometer using Co Kα radiation (λ = 1.790 Å) at 30 kV and 10 mA with 2° min^–1^ and steps of 0.01°.

### Operando Reactor

The experiments were performed at the APE-HE beamline of the Elettra Synchrotron in Trieste, with proposal no. 20205379. The beamline was equipped with an operando cell for gas phase reactions between a few mbar to 1 bar pressure.^30−32^ All spectra were collected in total electron yield (TEY) mode by measuring the drain current and applying a bias of 60 V between the sample and the nano-Si_3_N_4_ membrane separating the sample environment from the UHV chamber hosting the reaction cell. The Agilent 490 Micro Gas Chromatography system was employed to monitor the catalytical products occurring in the reactor during the operando XAS measurements.^31,32^

### Operando Soft XAS Measurements

The samples in the form of powders were mounted on titanium sample holders by mechanical compression and introduced in the operando reactor cell. The operando reactor was equipped with a gas line composed of three gas flowmeters, and the experiments were performed with a total flow of 50 mL·min^–1^ at 1 bar of total pressure, unless stated otherwise. The measurement procedure is as follows: the first step of the experiment consisted of a thermal treatment in a He (100%) atmosphere in order to remove the surface contaminants (step 1). In case of the LaMnO_3_, LaMn_0.5_Co_0.5_O_3_, LaMn_0.75_Co_0.25_O_3_, and LaCoO_3_ samples, the temperature of the samples was increased from room temperature to 350 °C under He. After cooling down to room temperature (step 2), except in the case of LaCoO_3_ which was cooled to 200 °C, similarly to previous experiments,^36^ the samples were heated again to the target temperature at a rate of 5 °C min^–1^ under a 12% CO plus O_2_ gas mixture (V_CO_:V_O2_ = 1) (step 3). At the same time, the gas chromatograms measured on the exhaust gas coming out from the reaction cell were acquired in order to analyze the reaction products. The measurements were performed after waiting for 20 min in the target temperature. Then, keeping the samples at the maximum temperature, the 6% CO/6% O_2_ mixture was removed and substituted with a 12% CO gas (step 4). The Co L_2,3_, Mn L_2,3_, La M_4,5_, and oxygen K-edge XAS have been recorded with ∼20 cycles of fast scan cycles with a photon energy step of 0.05 eV. The energy resolution was ∼0.3 eV full width at half-maximum (fwhm).

## 3 Theoretical Simulations

The 2p XAS spectra of Mn and Co have been calculated with charge transfer multiplet theory based on a cluster model of MO_6_ (M is Co or Mn and O is the ligand oxygen) using the *Quanty* program.^37−42^ We used the Anderson impurity model with two configurations on the basis of on-site Coulomb repulsion interaction U_dd_, ligand to metal change transfer Δ, symmetry-dependent hopping (V_t2g_ and V_eg_), and the core-hole potential (Q_2p_). We only consider two configurations because the third configuration does not visibly affect the 2p XAS spectra. The electron–electron interactions are parametrized with Slater integral parameters F_dd_^2^, F_dd_^4^, F_pd_^2^, G_pd_^1^, and G_pd_^3^ and are calculated from first-principles with the Cowan code.^43^ The configuration weights are calculated with CTM4XAS.^44^ The energy diagrams and ground-state projections are carried out with CTM4DOC.^45^ The temperature-dependent XAS calculations used a Boltzmann distribution over all low-lying configurations.^42^ Given that the sample is in powder form, all theoretical XAS calculations consider isotropic spectra summed over the different polarizations. The calculated photon energy is adjusted to match the experimental spectra.

## 4 Results

We start by showing the XAS spectra and data analysis of the four samples one by one. In the Discussion, we compare the main results from the samples in relation to their catalytic activity.

### 4.1 Heating of LaMnO_3_ in Helium Atmosphere

**Figure 1:**
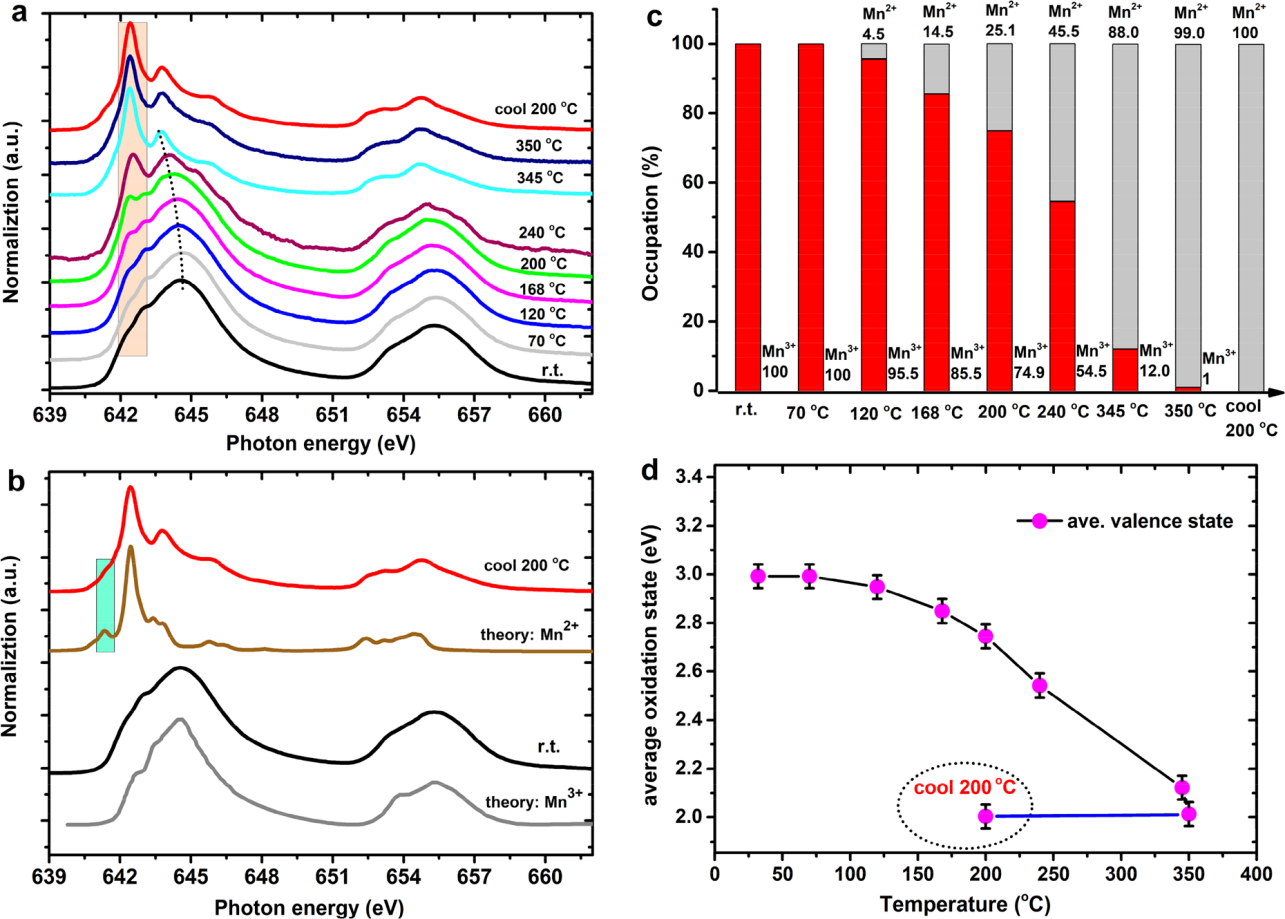
Pretreatment heating study of LaMnO_3_ in 1 bar He gas. (a) Mn L_2,3_ XAS spectra from room temperature to 350 °C and after cooling down to 200 °C. (b) Charge transfer multiplet simulations of Mn^3+^ (D_4h_) and Mn^2+^(O_h_) 2p XAS in comparison with the experiments. (c) The components of Mn^3+^ and Mn^2+^ over the measurements. (d) Temperature-dependent average oxidation state. All spectra have been measured with total electron yield and are normalized from 0 to 1.0, indicated with “normalization (to) arbitrary units (a.u.)”.

Figure 1 shows the pretreatment heating (step 1) of the LaMnO_3_ nanoparticles in 1 bar He atmosphere from room temperature (r.t. is 25 °C) to 350 °C. Figure 1a shows temperature-dependent Mn 2p XAS spectra, in which the Mn^2+^ character increases (indicated with an orange rectangle). Due to the surface carbon contaminants, we observe a reduction of the catalyst, where we note that electron yield probes the top 4 nm of the sample. Because of this surface reduction, we have pretreated all catalysts samples by heating in He. In case of the soft X-ray L-edges, the white line is much larger than the edge jump. In addition, there is background from the beamline plus potential saturation effects. This makes it usually unreliable to normalize the XAS edges to their edge jump.

Figure 1b shows charge transfer multiplet simulations of high-spin Mn^3+^ (3d^4^) 2p XAS in tetragonal (D_4h_) symmetry and high-spin Mn^2+^ (3d^5^) 2p XAS in octahedral (O_h_) symmetry. The related parameters for the calculations are explained in the Supporting Information (Table S2). Figure 1c shows the population of Mn^3+^ and Mn^2+^ over the measured temperature range, where we assume that the r.t. and 70 °C spectra are pure Mn^3+^ and the spectrum after cooling down to 200 °C is pure Mn^2+^. This is in agreement with the simulations in Figure 1b. We note that the Mn^2+^ spectrum shows saturation effects (indicated with the green rectangle in Figure 1b), which have also been observed in previous measurements;^46^ more details of the spectral fitting are given in the Supporting Information (Figure S3). Figure 1d shows the temperature-dependent average oxidation state graphically.

### 4.2 Operando CO Oxidation of LaMnO_3_

**Figure 2:**
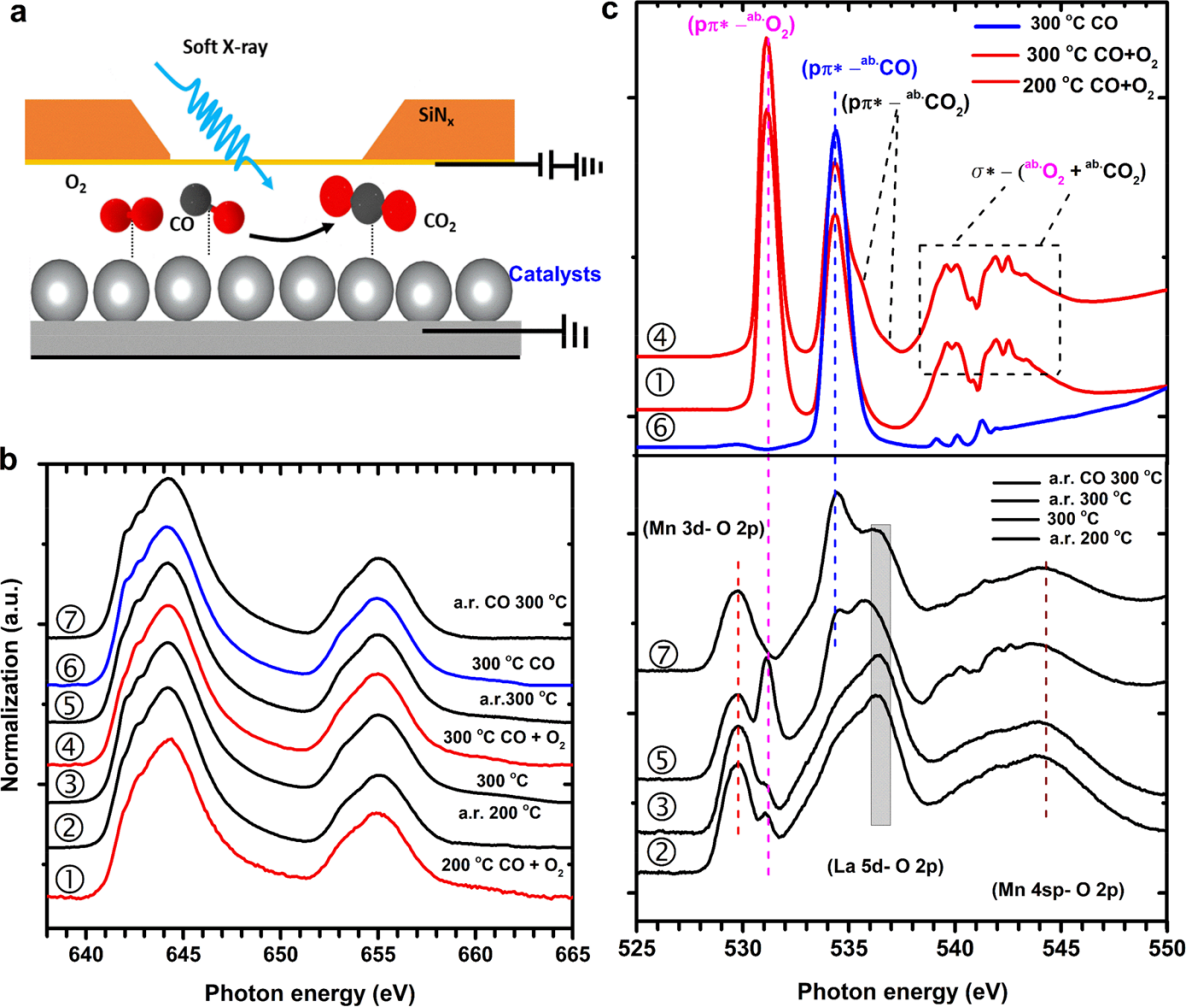
Operando Mn 2p and O 1s XAS in the CO oxidation using LaMnO_3_ catalyst. (a) The scheme of an operando reactor. (b) Operando Mn 2p XAS. (c) Operando O 1s XAS normalized from 0 to 1. The abbreviations rt and a.r. are room temperature and after reaction, respectively; the gray rectangle in the bottom panel indicates the La 5d–O 2p band. Labels ① to ⑦ indicate the temperature and gas conditions. If no gas conditions are indicated, the measurements are performed under helium. For clarity, we have also indicated each gas condition with the color of the line: black for helium, red for CO + O_2_, and blue for CO. Details are given in the Experimental Methods Section.

Figure 2a shows a scheme of the operando reactor, where the XAS spectrum is measured by TEY drain current.^30^Figure 2b shows the Mn L_2,3_-edge of LaMnO_3_ under the working condition of 6% CO/6% O_2_ in He at 1 bar total pressure. Spectrum ① is measured at 200 °C and was reoxidized to Mn^3+^. The spectral shape does not change (significantly), indicating that the oxidation state remains constant at Mn^3+^. It is known that LaMnO_3_ easily forms defects to become LaMnO_3+δ_, resulting in up to ∼5% Mn^2+^ during the heating/operando process. Figure 2c shows the oxygen K-edge XAS, where the spectra are a combination of the oxide catalyst and the gas phase oxygen species, both adsorbed and in the gas phase. Under the operando conditions of CO + O_2_ and CO, the oxygen K-edges are completely dominated by the gas phase spectra that are given in Figure 2c. Next to the CO and the O_2_ peaks, we also observe the CO_2_ peaks at 300 °C, indicating that the CO oxidation reaction is running. The oxygen K-edge of LaMnO_3_ contains oxygen p-character of the empty metal states, respectively, Mn 3d (528 to 531 eV), La 5d (532 to 539 eV), and Mn 4p (540 to 548 eV), as can also be shown with Density-Functional-Theory-based calculations.^18^

The oxygen K-edges that have been measured in helium atmosphere also show peaks related to (adsorbed) O_2_ possibly from air absorbed into the nanoparticle of the powder sample. The presence of the gas phase spectra make it difficult to determine the exact oxygen K edge spectra of the sample surface. In principle, one could subtract the spectra of O_2_ and CO, but in practice, this creates too much uncertainty to reliably determine potential small changes. As far as we could determine, no reliable visible changes can be determined in the oxygen K-edge of the surface. Because of this, we focus on the metal spectra in the remainder. The oxygen K-edge spectra of all other samples are given in the Supporting Information.

### 4.3 Operando CO Oxidation of LaMn_0.5_Co_0.5_O_3_

**Figure 3:**
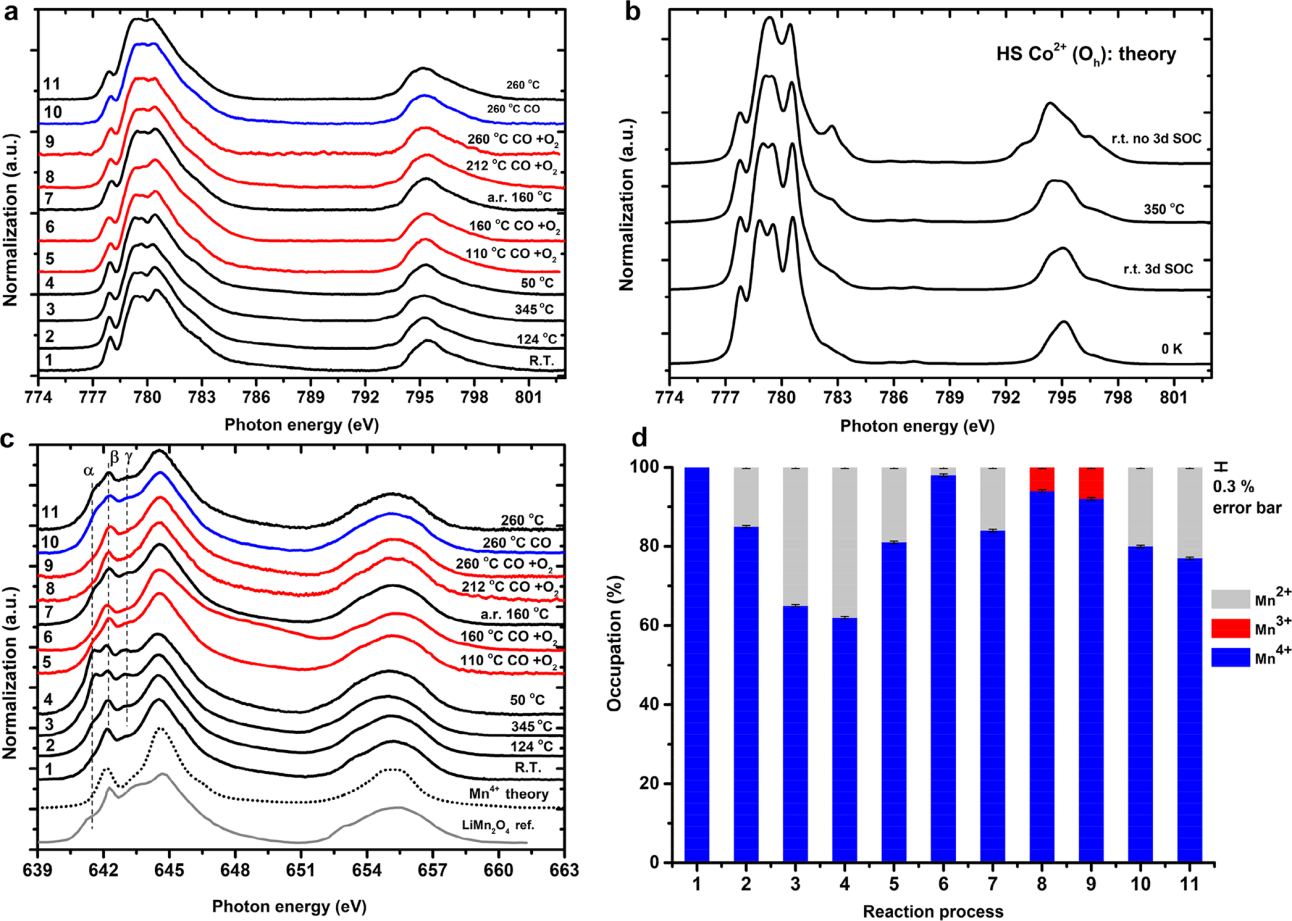
Operando Co and Mn 2p XAS of LaMn_0.5_Co_0.5_O_3_ in the CO oxidation. (a) Co 2p XAS under different temperatures with or without CO + O_2_ (1 bar). (b) Multiplet ligand field simulations of Co^2+^ 2p XAS. (c) Mn 2p XAS under different temperatures with or without CO + O_2_; the two bottom spectra are a Mn 2p XAS of LiMn_2_O_4_ reproduced with permission from ref (47), Copyright (1994, Elsevier), and a Mn^4+^ simulation. α indicates the position of the main peak of Mn^2+^, β indicates the first peak of Mn^4+^, and γ indicates the main peak of Mn^3+^. (d) Components of Mn^2+^, Mn^3+^, and Mn^4+^. Labels 1 to 11 indicate the temperature and gas conditions. If no gas conditions are indicated, the measurements are performed under helium. For clarity, we have also indicated each gas condition with the color of the line: black for helium, red for CO + O_2_, and blue for CO. Details are given in the Experimental Methods Section.

Figure 3 shows Co and Mn 2p XAS of the LaMn_0.5_Co_0.5_O_3_ nanoparticles. The Co 2p XAS (Figure 3a) shows only minor changes during heating and under operando CO + O_2_ catalytic conditions. Figure 3b shows crystal field multiplet calculations of Co^2+^. The 3d^7^ high-spin ^4^T_1_ ground state is changing with temperature due to the increased occupation of the (3d spin–orbit split) ^4^T_1_ state. Apart from this temperature-induced effect, the Co L_2,3_-edge does not change, indicating that the Co sites remain high-spin ^4^T_1_ under all conditions. Minor spectral variations can be due to small (averaged) symmetry distortions that can slightly change the spectral shape.

Figure 3c shows the Mn 2p XAS spectra. The room temperature spectrum can be reproduced from an octahedral Mn^4+^ site. From r.t. to 345 °C and then cooling to 50 °C, Mn^4+^ gradually reduces to low oxidation state (Mn^3+^ and Mn^2+^). We performed linear combination fitting using Mn^2+^, Mn^3+^, and Mn^4+^ 2p XAS reference spectra to determine the components (Figure 3d). The details are given in the Supporting Information Figures S4–S5. Heating in helium slowly increases the amount of Mn^2+^ (conditions 1 to 4).The measurements in CO + O_2_ up to 160 °C reoxidize the system to Mn^4+^. Operando measurements up to 212 °C create a combination of Mn^3+^ and Mn^4+^. Switching to pure CO + He reduces Mn again to partial Mn^2+^. In conclusion, we observe that under operando reaction conditions, Co remains 2+ and the amount of Mn^4+^ increases.

### 4.4 Operando CO Oxidation of LaMn_0.75_Co_0.25_O_3_

**Figure 4:**
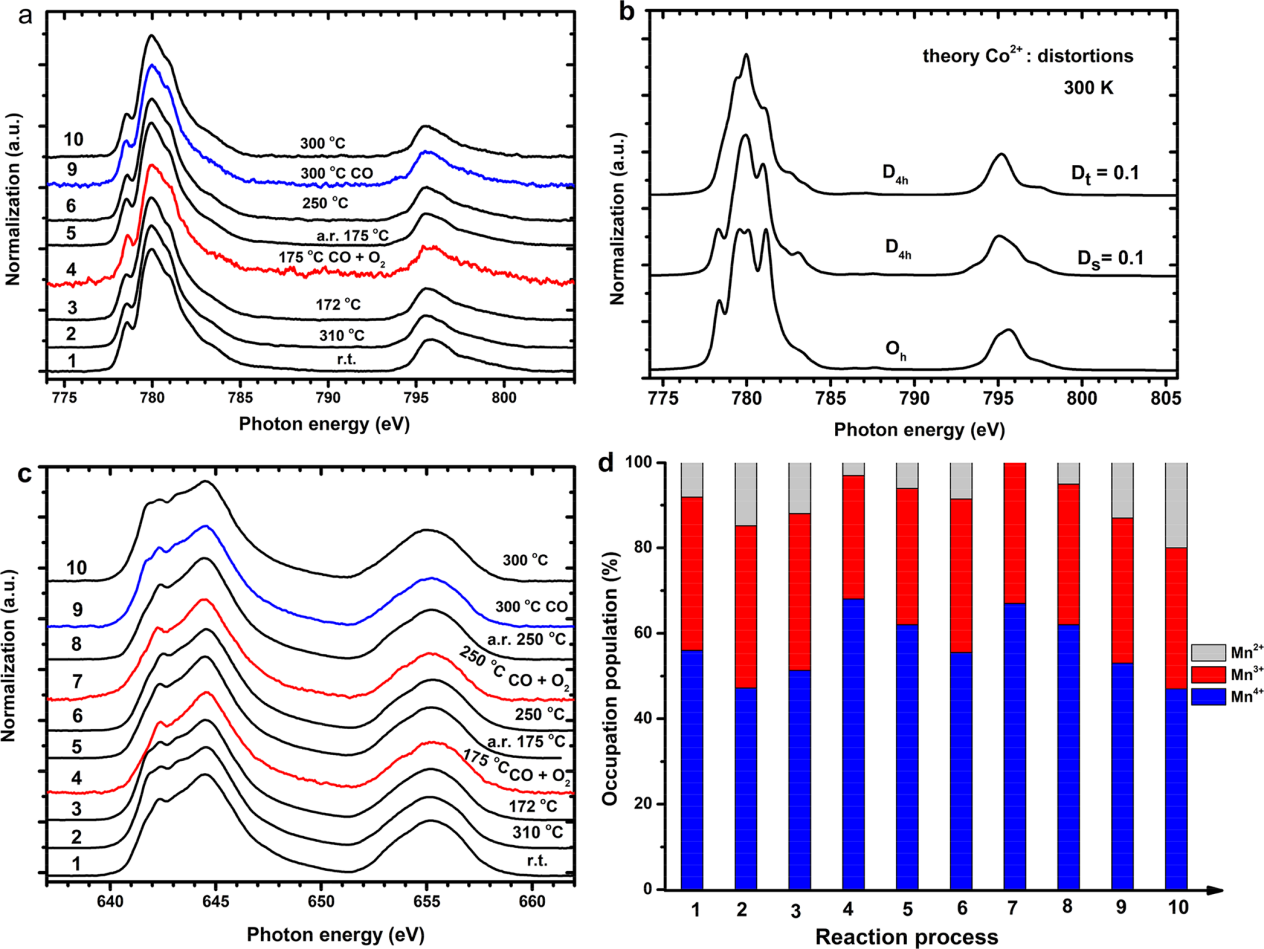
Operando Co and Mn 2p XAS of LaMn_0.75_Co_0.25_O_3_ in the CO oxidation. (a) Co 2p XAS under different conditions: temperature and CO + O_2_ gas. (b) The multiplet ligand field simulation of Co^2+^ 2p XAS by using a tetragonal symmetry distortion at 300 K. (c) Operando Mn 2p XAS under different conditions. (d) The occupations of Mn^2+^, Mn^3+^, and Mn^4+^ over the reaction process. Labels 1 to 10 indicate the temperature and gas conditions. If no gas conditions are indicated, the measurements are performed under helium. For clarity, we have also indicated each gas condition with the color of the line: black for helium, red for CO + O_2_, and blue for CO. Details are given in the Experimental Methods Section.

Figure 4a shows the Co 2p XAS spectra of LaMn_0.75_Co_0.25_O_3_, which can be interpreted as Co^2+^, similar to LaMn_0.5_Co_0.5_O_3_. The detailed spectral shape is different from LaMn_0.5_Co_0.5_O_3_ (Figure 3a) due to a symmetry reduction of the octahedral Co site to a tetragonal symmetry, which is likely caused by the dominance of the Mn^3+^ sites being Jahn–Teller ions. The tetragonal symmetry has been simulated in Figure 4b (more details in the Supporting Information Figure S6). The related parameters of D_s_ and D_t_ effectively cause a broadening of the spectral shape. Figure 4c shows the Mn 2p XAS, in which the spectral variations indicate a combination of Mn^4+^, Mn^3+^, and Mn^2+^ valences. The details of the fitting are given in the Supporting Information Figures S7–S8. The heating effects are sensitive to the Mn^2+^–Mn^4+^ components (Figure 4d). Operando CO + O_2_ conditions at both 175 and 260 °C increase the Mn^4+^ and reduce the Mn^2+^ components.

### 4.5 Operando CO Oxidation of LaCoO_3_

**Figure 5:**
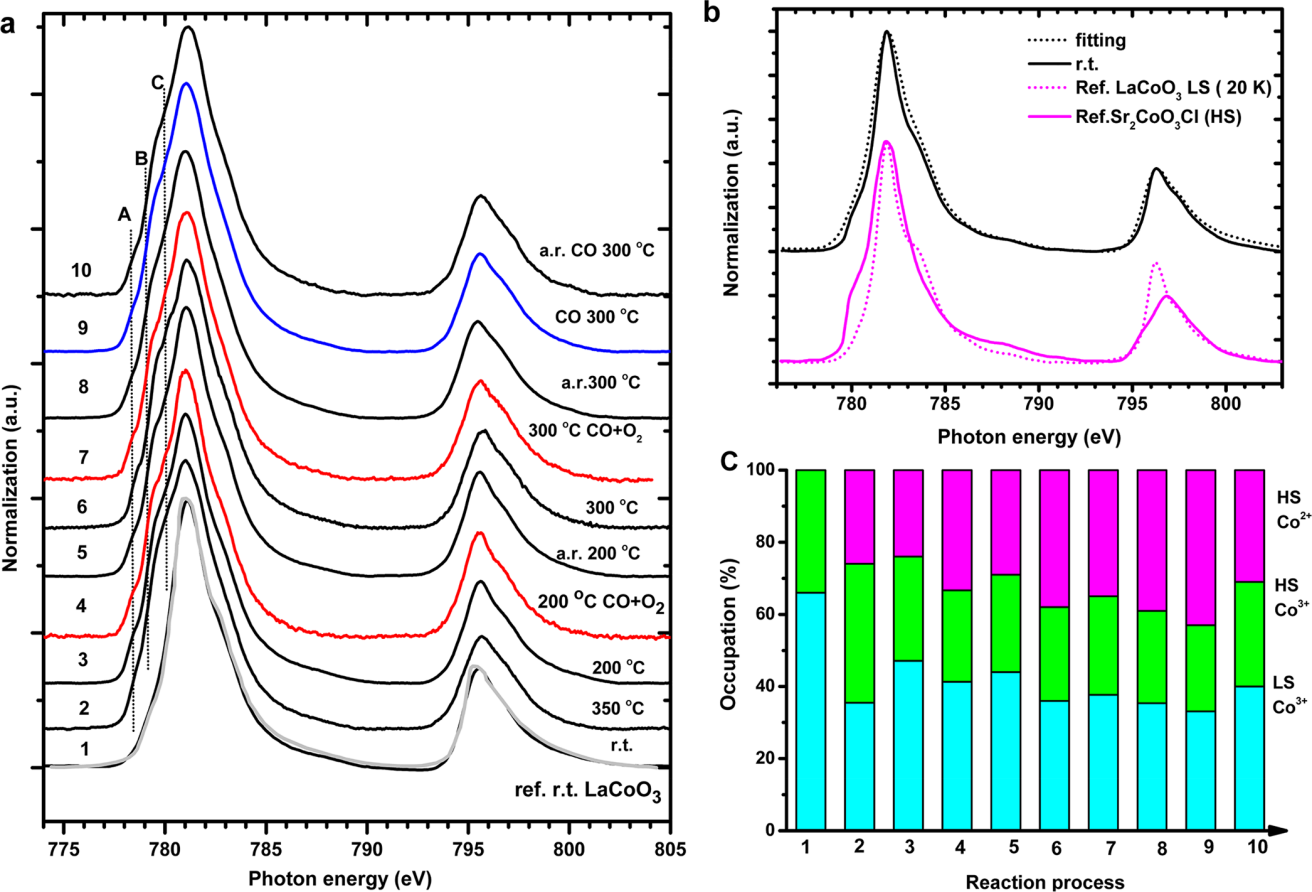
(a) The operando Co 2p XAS of LaCoO_3_ for CO oxidation. The reaction process is from bottom to top, with reference r.t. LaCoO_3_ (gray) single crystal reproduced with permission from ref (20). Copyright (2006, American Physical Society). (b) r.t. Co 2p XAS fitting by pure low-spin Co^3+^ of LaCoO_3_ (20 K) reproduced with permission from ref (20). Copyright (2006, American Physical Society) + high-spin Co^3+^ of Sr_2_CoO_3_Cl reproduced with permission from ref (49). Copyright (2009, American Physical Society). (c) The population of high-spin and low-spin Co^3+^ and Co^2+^ during the reaction process from panel (a). Labels 1 to 10 indicate the temperature and gas conditions. If no gas conditions are indicated, the measurements are performed under helium. For clarity, we have also indicated each gas condition with the color of the line: black for helium, red for CO + O_2_, and blue for CO. Details are given in the Experimental Methods Section.

Figure 5a shows the operando Co L_2,3_ XAS spectra of LaCoO_3_. The labels “A”, “B”, and “C” indicate Co^2+^ features. Figure 5b shows the r.t. Co 2p XAS linear combination Co^3+^ high-spin/low-spin fitting. The treatment of LaCoO_3_ was different from the other three samples in the sense that the sample was not cooled to room temperature after the initial heat treatment in helium. Because Co^3+^ is known to exist in both high-spin and low-spin in LaCoO_3_ system, including a temperature-dependence in single crystal LaCoO_3_,^20,48^ we used pure low-spin Co^3+^ of LaCoO_3_ (20 K)^20^ and high-spin Co^3+^ of Sr_2_CoO_3_Cl^49^ for the linear combination fitting at room temperature. Co 2p XAS shows a 34% high-spin Co^3+^ occupation, 66% low-spin Co^3+^, and no Co^2+^ component. We note that the LaCoO_3_ nanoparticle is a bit different from single crystals as measured by Haverkort et al.^20^ and Tomiyasu et al.^48^ In order to quantitatively estimate the occupations of high-spin and low-spin and the presence of Co^2+^ above r.t., we focus on the L_2_-edge and: (i) determine the spin ratio by linear combination from reference low-spin Co^3+^ of LaCoO_3_ and high-spin Co^3+^ of Sr_2_CoO_3_Cl; (ii) assume that the same temperature has the same low-spin/high-spin ratio under operando conditions; (iii) the temperature-dependent high-spin Co^2+^ was used as the Co^2+^ 2p spectra reference taken from Figure 3a to minimize thermal effects. All L_2_-edge fittings are given in Supporting Information Figures S10–S13, resulting in the numbers given in Figure 5c. Both operando 200 and 300 °C spectra show similar results for the presence of Co^2+^.

## 5 Discussion

Table 1 shows the main observations regarding the averages valences of Mn and Co, where (*cond.6*) indicates the spectrum number 6. The symmetry (O_h_ vs D_4h_) and spin state (% HS) of the four measured LaMn_1–*x*_Co_*x*_O_3_ catalysts for the CO oxidation reaction are provided. The activity, measured as the CO_2_ production, is normalized to 1.0 for the highest activity of LaMn_0.5_Co_0.5_O_3_. We observe CO_2_ being formed from the oxygen K-edge at 300 °C (Figure 2c and Figure S15–S16) and the CO_2_ products in Figure S18 (Supporting Information), which confirms the operando CO oxidation reaction.

**Table 1:** Main Observations of the Catalytic Activity, the Average Valences of ⟨Mn⟩ and ⟨Co⟩, and the Symmetry and the Spin State of Mn and Co Ions of LaMn_1–*x*_Co_*x*_O_3_ Samples

Sample	⟨Mn⟩ Helium	⟨Mn⟩ Operando	⟨Co⟩ Helium	⟨Co⟩ Operando	Activity (normalized)
LaMnO_3_	3.00	2.97	–	–	0.2
LaMn_0.75_Co_0.25_O_3_	3.43 (*cond.6*)	3.67 (*cond.7*)	2.0 (D_4h_)	2.0	0.3
LaMn_0.5_Co_0.5_O_3_	3.64 (*cond.7*)	3.92 (*cond.9*)	2.0 (O_h_)	2.0	1.0
LaCoO_3_	–	–	3.0 (35% HS)	2.65 (63% HS)	0.2

In LaMnO_3_, the valence state of Mn is 3+ under helium, and it does not change much under reaction conditions. Both LaMn_0.5_Co_0.5_O_3_ and LaMn_0.75_Co_0.25_O_3_ catalysts show mixtures of the Mn valences, where Mn^4+^ increases and Mn^2+^ decreases during the operando CO + O_2_ oxidation, indicating that Mn^4+^ is the active site. In LaMn_0.75_Co_0.25_O_3_, the average valence increases to 3.67, and in LaMn_0.5_Co_0.5_O_3_, it increases to 3.92, indicating that a larger amount of Mn^4+^ increases the activity. Cobalt remains 2+ in LaMn_0.5_Co_0.5_O_3_ and LaMn_0.75_Co_0.25_O_3_. In LaMn_0.75_Co_0.25_O_3_, the Co^2+^ site has tetragonal (D_4h_) symmetry, likely induced by the dominance of the LaMnO_3_ structure. The Co^2+^ site is octahedral in LaMn_0.5_Co_0.5_O_3_, similar to the LaCoO_3_ structure. In LaCoO_3_, we observe fluctuations between (high-spin) Co^2+^ and mixed high-spin/low-spin Co^3+^. We describe the increase in Co^2+^ under reaction conditions to the compensation of the valence increase of Mn. Cobalt thus allows manganese to reach a higher average valence, which drastically increases the activity.

Unfortunately, analysis of the oxygen K-edge was hampered by the background of the gas phase signal. Future efforts to better separate the oxygen surface signal from the gas phase signal will improve this situation and drastically improve the options to also use the full strength of the oxygen K-edge spectra.

## 6 Conclusions

We have investigated operando soft X-ray Mn and Co 2p XAS as well as O 1s XAS spectra of LaMn_1–*x*_Co_*x*_O_3_ nanoparticles for CO oxidation. Based on charge transfer multiplet calculations, we quantitatively identified the valence and spin states of Co and Mn. LaMn_0.5_Co_0.5_O_3_ has the highest activity attributed to octahedral Co^2+^ combining with (almost pure) Mn^4+^. LaMn_0.75_Co_0.25_O_3_ has the second highest activity, which is caused by the combination of its LaMnO_3_ structure (shown by the tetragonal Co^2+^ sites) and its high average valence (under reaction conditions) of 3.67. LaMnO_3_ and LaCoO_3_ are much less active due to the absence of Mn^4+^ (or Co^4+^) sites. We have shown that operando soft X-ray XAS are very effective to study the electronic structure of the (near) surface states due to the combination of ∼4 nm probing depth and the sharp soft X-ray XAS spectra into the 3d states, which are very sensitive to details of the electronic structure.

## Supporting Information Available

Experimental procedure scheme, XRD, Co–Mn 2p XAS fitting, Co^2+^ 2p XAS calculation in D_4h_ distortion and HF value effects, Mn^2+^ 2p XAS simulation at 20–1000 K, operando O 1s XAS, XAS simulation parameters in charge transfer multiplets (PDF)

## References

[ref1] Yang F.; Graciani J.; Evans J.; Liu P.; Hrbek J.; Sanz J. F.; Rodriguez J. A. CO Oxidation on Inverse CeO_x_/Cu(111) Catalysts: High Catalytic Activity and Ceria-Promoted Dissociation of O_2_. J. Am. Chem. Soc. 2011, 133 (10), 3444–3451. 10.1021/ja1087979. 21341793

[ref2] Wang Y.; Ren P.; Hu J.; Tu Y.; Gong Z.; Cui Y.; Zheng Y.; Chen M.; Zhang W.; Ma C.; Yu L.; Yang F.; Wang Y.; Bao X.; Deng D. Electron Penetration Triggering Interface Activity of Pt-Graphene for CO Oxidation at Room Temperature. Nat. Commun. 2021, 12 (1), 5814 10.1038/s41467-021-26089-y. 34608162 PMC8490350

[ref3] Zhang Z.; Tian J.; Lu Y.; Yang S.; Jiang D.; Huang W.; Li Y.; Hong J.; Hoffman A. S.; Bare S. R.; Engelhard M. H.; Datye A. K.; Wang Y. Memory-Dictated Dynamics of Single-Atom Pt on CeO_2_ for CO Oxidation. Nat. Commun. 2023, 14, 2664 10.1038/s41467-023-37776-3. 37160890 PMC10169862

[ref4] Imanaka N.; Masui T.; Imadzu H.; Yasuda K. Carbon Monoxide Oxidation at Room Temperature on Pt/CeO_2_- ZrO_2_-Bi_2_O_3_ Catalysts. Chem. Commun. 2011, 47 (39), 11032–11034. 10.1039/c1cc12348c. 21909520

[ref5] Peña M. A.; Fierro J. L. G. Chemical Structures and Performance of Perovskite Oxides. Chem. Rev. 2001, 101 (7), 1981–2018. 10.1021/cr980129f. 11710238

[ref6] Simböck J.; Ghiasi M.; Schönebaum S.; Simon U.; de Groot F. M. F.; Palkovits R. Electronic Parameters in Cobalt-Based Perovskite-Type Oxides as Descriptors for Chemocatalytic Reactions. Nat. Commun. 2020, 11 (1), 652 10.1038/s41467-020-14305-0. 32005805 PMC6994687

[ref7] Zhu H.; Zhang P.; Dai S. Recent Advances of Lanthanum-Based Perovskite Oxides for Catalysis. ACS Catal. 2015, 5 (11), 6370–6385. 10.1021/acscatal.5b01667.

[ref8] Wang Y.; Ren J.; Wang Y.; Zhang F.; Liu X.; Guo Y.; Lu G. Nanocasted Synthesis of Mesoporous LaCoO_3_ Perovskite with Extremely High Surface Area and Excellent Activity in Methane Combustion. J. Phys. Chem. C 2008, 112 (39), 15293–15298. 10.1021/jp8048394.

[ref9] Lu H.; Zhang P.; Qiao Z. A.; Zhang J.; Zhu H.; Chen J.; Chen Y.; Dai S. Ionic Liquid-Mediated Synthesis of Meso-Scale Porous Lanthanum-Transition-Metal Perovskites with High CO Oxidation Performance. Chem. Commun. 2015, 51 (27), 5910–5913. 10.1039/C5CC00534E. 25727232

[ref10] Park J.; Cheong S. W.; Chen C. Double-Exchange Ferromagnetism in L. Phys. Rev. B 1997, 55 (17), 11072–11075. 10.1103/PhysRevB.55.11072.

[ref11] Burnus T.; Hu Z.; Hsieh H. H.; Joly V. L. J.; Joy P. A.; Haverkort M. W.; Wu H.; Tanaka A.; Lin H. J.; Chen C. T.; Tjeng L. H. Local Electronic Structure and Magnetic Properties of LaMn_0.5_Co_0.5_O_3_ Studied by x-Ray Absorption and Magnetic Circular Dichroism Spectroscopy. Phys. Rev. B 2008, 77 (12), 125124 10.1103/PhysRevB.77.125124.

[ref12] Bonn M.; Funk S.; Hess C.; Denzler D. N.; Stampfl C.; Scheffler M.; Wolf M.; Ertl G. Phonon- versus electron-mediated desorption and oxidation of CO on Ru(0001). Science 1999, 285 (5430), 1042–1045. 10.1126/science.285.5430.1042. 10446045

[ref13] Freund H. J.; Meijer G.; Scheffler M.; Schlögl R.; Wolf M. CO oxidation as a prototypical reaction for heterogeneous processes. Angew. Chem., Int. Ed. 2011, 50 (43), 10064–10094. 10.1002/anie.201101378. 21960461

[ref14] Suntivich J.; Gasteiger H. A.; Yabuuchi N.; Nakanishi H.; Goodenough J. B.; Shao-Horn Y. Design Principles for Oxygen-Reduction Activity on Perovskite Oxide Catalysts for Fuel Cells and Metal–Air Batteries. Nat. Chem. 2011, 3 (7), 546–550. 10.1038/nchem.1069. 21697876

[ref15] Suntivich J.; May K. J.; Gasteiger H. A.; Goodenough J. B.; Shao-Horn Y. A Perovskite Oxide Optimized for Oxygen Evolution Catalysis from Molecular Orbital Principles. Science (1979) 2011, 334 (6061), 1383–1385. 10.1126/science.1212858. 22033519

[ref16] Hwang J.; Rao R. R.; Giordano L.; Katayama Y.; Yu Y.; Shao-Horn Y. Perovskites in Catalysis and Electrocatalysis. Science 2017, 358 (6364), 751–756. 10.1126/science.aam7092. 29123062

[ref17] Mueller D. N.; MacHala M. L.; Bluhm H.; Chueh W. C. Redox Activity of Surface Oxygen Anions in Oxygen-Deficient Perovskite Oxides during Electrochemical Reactions. Nat. Commun. 2015, 6, 6097 10.1038/ncomms7097. 25598003

[ref18] Frati F.; Hunault M. O. J. Y.; De Groot F. M. F. Oxygen K-Edge X-Ray Absorption Spectra. Chem. Rev. 2020, 120 (9), 4056–4110. 10.1021/acs.chemrev.9b00439. 32275144 PMC7227067

[ref19] Hong W. T.; Stoerzinger K. A.; Lee Y. L.; Giordano L.; Grimaud A.; Johnson A. M.; Hwang J.; Crumlin E. J.; Yang W.; Shao-Horn Y. Charge-Transfer-Energy-Dependent Oxygen Evolution Reaction Mechanisms for Perovskite Oxides. Energy Environ. Sci. 2017, 10 (10), 2190–2200. 10.1039/C7EE02052J.

[ref20] Haverkort M. W.; Hu Z.; Cezar J. C.; Burnus T.; Hartmann H.; Reuther M.; Zobel C.; Lorenz T.; Tanaka A.; Brookes N. B.; Hsieh H. H.; Lin H. J.; Chen C. T.; Tjeng L. H. Spin State Transition in LaCoO_3_ Studied Using Soft X-Ray Absorption Spectroscopy and Magnetic Circular Dichroism. Phys. Rev. Lett. 2006, 97 (17), 176405 10.1103/PhysRevLett.97.176405. 17155490

[ref21] Royer S.; Duprez D.; Can F.; Courtois X.; Batiot-Dupeyrat C.; Laassiri S.; Alamdari H. Perovskites as Substitutes of Noble Metals for Heterogeneous Catalysis: Dream or Reality. Chem. Rev. 2014, 114 (20), 10292–10368. 10.1021/cr500032a. 25253387

[ref22] Autret C.; Hejtmánek J.; Knížek K.; Maryško M.; Jirák Z.; Dlouhá M.; Vratislav S. Electric Transport and Magnetic Properties of Perovskites LaMn_1-x_Co_x_O_3_ up to 900 K. J. Condens. Matter Phys. 2005, 17, 1601–1616. 10.1088/0953-8984/17/10/015.

[ref23] Sawada H.; Morikawa Y.; Hamada N.; Terakura K. Jahn-Teller Distortion and Magnetic Structures in LaMnO_3_. J. Magn. Magn. Mater. 1998, 177–181, 879–880. 10.1016/S0304-8853(97)00451-4.

[ref24] Hashimoto T.; Ishibashi S.; Terakura K. Jahn-Teller Distortion and Magnetic Structure in LaMnO_3_: A First-Principles Theoretical Study with Full Structure Optimizations. Phys. Rev. B 2010, 82 (4), 045124 10.1103/PhysRevB.82.045124.

[ref25] Sato K.; Matsuo A.; Kindo K.; Kobayashi Y.; Asai K. Field Induced Spin-State Transition in LaCoO_3_. J. Phys. Soc. Jpn. 2009, 78 (9), 093702 10.1143/JPSJ.78.093702.

[ref26] Asai K.; Yokokura O.; Suzuki M.; Naka T.; Matsumoto T.; Takahashi H.; Môri N.; Kohn K. Pressure Dependence of the 100 K Spin-State Transition in LaCoO_3_. J. Phys. Soc. Jpn. 1997, 66 (4), 967–970. 10.1143/JPSJ.66.967.

[ref27] Wang R. P.; Geessinck J.; Elnaggar H.; Birkhölzer Y. A.; Tomiyasu K.; Okamoto J.; Liu B.; Du C. H.; Huang D. J.; Koster G.; De Groot F. M. F. Low-Energy Orbital Excitations in Strained LaCoO_3_ Films. Phys. Rev. B 2019, 100 (16), 1–9. 10.1103/PhysRevB.100.165148.

[ref28] Braglia L.; Fracchia M.; Ghigna P.; Minguzzi A.; Meroni D.; Edla R.; Vandichel M.; Ahlberg E.; Cerrato G.; Torelli P. Understanding Solid-Gas Reaction Mechanisms by Operando Soft X-Ray Absorption Spectroscopy at Ambient Pressure. J. Phys. Chem. C 2020, 124 (26), 14202–14212. 10.1021/acs.jpcc.0c02546. PMC8008446 33815647

[ref29] Tavani F.; Fracchia M.; Tofoni A.; Braglia L.; Jouve A.; Morandi S.; Manzoli M.; Torelli P.; Ghigna P.; D’Angelo P. Structural and Mechanistic Insights into Low-Temperature CO Oxidation over a Prototypical High Entropy Oxide by Cu L-Edge Operando Soft X-Ray Absorption Spectroscopy. Phys. Chem. Chem. Phys. 2021, 23 (46), 26575–26584. 10.1039/D1CP03946F. 34812450

[ref30] Braglia L.; Tavani F.; Mauri S.; Edla R.; Krizmancic D.; Tofoni A.; Colombo V.; D’Angelo P.; Torelli P. Catching the Reversible Formation and Reactivity of Surface Defective Sites in Metal-Organic Frameworks: An Operando Ambient Pressure-NEXAFS Investigation. J. Phys. Chem. Lett. 2021, 12 (37), 9182–9187. 10.1021/acs.jpclett.1c02585. 34528795 PMC9282676

[ref31] Mauri S.; D’Olimpio G.; Ghica C.; Braglia L.; Kuo C. N.; Istrate M. C.; Lue C. S.; Ottaviano L.; Klimczuk T.; Boukhvalov D. W.; Politano A.; Torelli P. Hydrogen Production Mechanism in Low-Temperature Methanol Decomposition Catalyzed by Ni_3_Sn_4_ Intermetallic Compound: A Combined Operando and Density Functional Theory Investigation. J. Phys. Chem. Lett. 2023, 14 (5), 1334–1342. 10.1021/acs.jpclett.2c03471. 36727689

[ref32] Castán-Guerrero C.; Krizmancic D.; Bonanni V.; Edla R.; Deluisa A.; Salvador F.; Rossi G.; Panaccione G.; Torelli P. A Reaction Cell for Ambient Pressure Soft X-Ray Absorption Spectroscopy. Rev. Sci. Instrum. 2018, 89 (5), 054101 10.1063/1.5019333. 29864817

[ref33] Liu H.; Moré R.; Grundmann H.; Cui C.; Erni R.; Patzke G. R. Promoting Photochemical Water Oxidation with Metallic Band Structures. J. Am. Chem. Soc. 2016, 138 (5), 1527–1535. 10.1021/jacs.5b10215. 26771537

[ref34] Ghiasi M.; Delgado-Jaime M. U.; Malekzadeh A.; Wang R. P.; Miedema P. S.; Beye M.; De Groot F. M. F. Mn and Co Charge and Spin Evolutions in LaMn_1-X_Co_x_O_3_ Nanoparticles. J. Phys. Chem. C 2016, 120 (15), 8167–8174. 10.1021/acs.jpcc.6b00949.

[ref35] Niwa E.; Uematsu C.; Miyashita E.; Ohzeki T.; Hashimoto T. Conductivity and Sintering Property of LaNi_1–x_Fe_x_O_3_ Ceramics Prepared by Pechini Method. Solid State Ion. 2011, 201 (1), 87–93. 10.1016/j.ssi.2011.08.004.

[ref36] Fracchia M.; Ghigna P.; Pozzi T.; Anselmi Tamburini U.; Colombo V.; Braglia L.; Torelli P. Stabilization by Configurational Entropy of the Cu(II) Active Site during CO Oxidation on Mg_0.2_Co_0.2_Ni_0.2_Cu_0.2_Zn_0.2_O. J. Phys. Chem. Lett. 2020, 11 (9), 3589–3593. 10.1021/acs.jpclett.0c00602. 32309955 PMC8007101

[ref37] Lu Y.; Höppner M.; Gunnarsson O.; Haverkort M. W. Efficient Real-Frequency Solver for Dynamical Mean-Field Theory. Phys. Rev. B 2014, 90 (8), 085102 10.1103/PhysRevB.90.085102.

[ref38] Haverkort M. W.; Sangiovanni G.; Hansmann P.; Toschi A.; Lu Y.; Macke S. Bands, Resonances, Edge Singularities and Excitons in Core Level Spectroscopy Investigated within the Dynamical Mean-Field Theory. Euro Phys. Lett. 2014, 108 (5), 57004 10.1209/0295-5075/108/57004.

[ref39] Haverkort M. W. Quanty for Core Level Spectroscopy - Excitons, Resonances and Band Excitations in Time and Frequency Domain. J. Phys.: Conf. Ser. 2016, 712, 012001 10.1088/1742-6596/712/1/012001.

[ref40] Haverkort M. W.; Zwierzycki M.; Andersen O. K. Multiplet Ligand-Field Theory Using Wannier Orbitals. Phys. Rev. B 2012, 85 (16), 165113 10.1103/PhysRevB.85.165113.

[ref41] Retegan M. Crispy, v0.7.3, 2019.

[ref42] De Groot F.; Kotani A. Core Level Spectroscopy of Solids; CRC Press, 2008.

[ref43] Cowan R. D. The Theory of Atomic Structure and Spectra; University of California Press: Berkeley, 1981.

[ref44] Stavitski E.; de Groot F. M. F. The CTM4XAS Program for EELS and XAS Spectral Shape Analysis of Transition Metal L Edges. Micron 2010, 41 (7), 687–694. 10.1016/j.micron.2010.06.005. 20637641

[ref45] Delgado-Jaime M. U.; Zhang K.; Vura-Weis J.; De Groot F. M. F. CTM4DOC: Electronic Structure Analysis from X-Ray Spectroscopy. J. Synchrotron Radiat. 2016, 23, 1264–1271. 10.1107/S1600577516012443. 27577785 PMC5006656

[ref46] Ghiringhelli G.; Matsubara M.; Dallera C.; Fracassi F.; Tagliaferri A.; Brookes N. B.; Kotani A.; Braicovich L. Resonant Inelastic X-Ray Scattering of MnO: L_2,3_ Edge Measurements and Assessment of Their Interpretation. Phys. Rev. B 2006, 73 (3), 035111 10.1103/PhysRevB.73.035111.

[ref47] De Groot F. M. F. X-Ray Absorption and Dichroism of Transition Metals and Their Compounds. J. Electron Spectrosc. Relat. Phenom. 1994, 67 (4), 529–622. 10.1016/0368-2048(93)02041-J.

[ref48] Tomiyasu K.; Okamoto J.; Huang H. Y.; Chen Z. Y.; Sinaga E. P.; Wu W. B.; Chu Y. Y.; Singh A.; Wang R.-P.; de Groot F. M. F.; Chainani A.; Ishihara S.; Chen C. T.; Huang D. J. Coulomb Correlations Intertwined with Spin and Orbital excitations in LaCoO_3_. Phys. Rev. Lett. 2017, 119 (19), 196402 10.1103/PhysRevLett.119.196402. 29219525

[ref49] Chang C. F.; Hu Z.; Wu H.; Burnus T.; Hollmann N.; Benomar M.; Lorenz T.; Tanaka A.; Lin H.-J.; Hsieh H. H.; Chen C. T.; Tjeng L. H. Spin Blockade, Orbital Occupation, and Charge Ordering in La_1.5_Sr_0.5_CoO_4_. Phys. Rev. Lett. 2009, 102 (11), 116401 10.1103/PhysRevLett.102.116401. 19392219

