# Supplementary material for: Operando Soft X-ray Absorption of LaMn1–xCoxO3 Perovskites for CO Oxidation
Source: ACS Catal. 2024 Jul 12;14(15):11243–51. doi: 10.1021/acscatal.4c03259 (PMC11301621; doi:10.1021/acscatal.4c03259)
Supplement: Supplementary file 1 — cs4c03259_si_001.pdf [file cs4c03259_si_001.pdf]

***Operando soft X-ray absorption of $LaMn_{1-x}Co_xO_3$ perovskites for CO oxidation***

Qijun Che[1*], Mahnaz Ghiasi[1], Luca Braglia[2], Matt L. J. Peerlings[1], Silvia Mauri[3], Piero Torelli[3], Petra de Jongh[1], Frank M. F. de Groot[1, *]

**Address**

*[1]Materials Chemistry and Catalysis, Debye Institute for Nanomaterials Science, Utrecht University, Universiteitsweg 99, 3584 CG Utrecht, The Netherlands*

*[2]AREA Science Park, Padriciano 99, I-34149 Trieste, Italy*

*[3]CNR-Istituto Officina dei Materiali, 34149 Trieste, Italy*

*[*]Corresponding authors: q.che@uu.nl; F.M.F.deGroot@uu.nl*

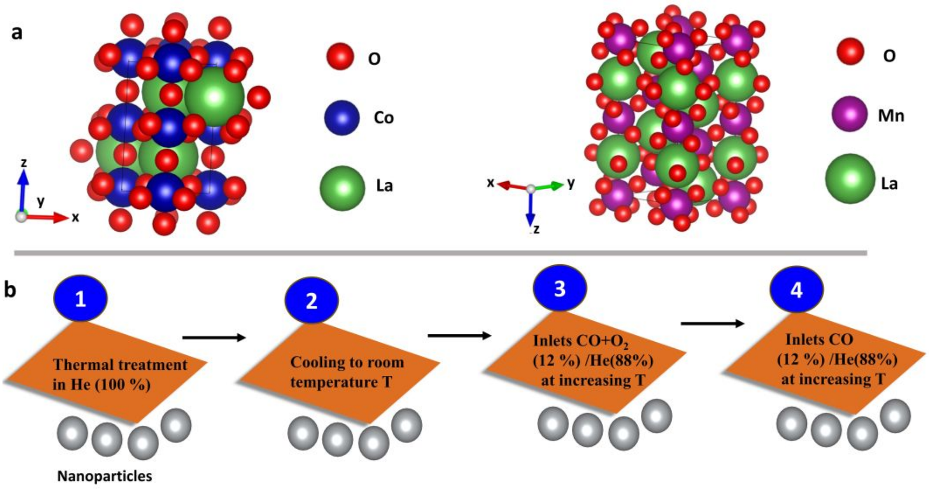


Figure S1. (a) The crystal structures of single monoclinic $LaCoO_3$ and rhombohedral $LaMnO_3$. (b) Experimental procedure[1] employed during the soft x-ray operando XAS measurements.

**XRD study**

The wide-angle XRD pattern of the $LaMn_{1-x}Co_xO_3$ perovskites are shown in Fig. S2. $LaMnO_3$ shows a rhombohedral structure (PDF no. 04-012-5560) with space group R-3c (167), and $LaCoO_3$ exhibits a single monoclinic phase (PDF no. 04-014-5176) with space group I2/a (15). With x from 0.25 to 0.75, the structure of the sample gradually evolved from the rhombohedral phase to the monoclinic phase. For samples with x≤0.5, the structural phase is close to the rhombohedral case. In case of $LaMn_{0.5}Co_{0.5}O_3$, the satellite peak at ~55.7$^o$ can be assigned to the monoclinic phase with a crystal face of (0 0 4). With x > 0.5, the structural phases of the samples change into monoclinic, with a few rhombohedral features remaining. The $LaMn_{0.5}Co_{0.75}O_3$ sample is nearest to the monoclinic structure with respect to the $LaCoO_3$ structure, but the peak located at ~54.9 $^o$ shows the rhombohedral character with the (0 2 4) crystalline plane. The smaller peak at ~34.5 $^o$ can be ascribed to $La_2O_3$.[2] Additionally, we calculated the crystal sizes using the Debye-Scherrer equation as 20 nm to 40 nm (cf. Table S1).

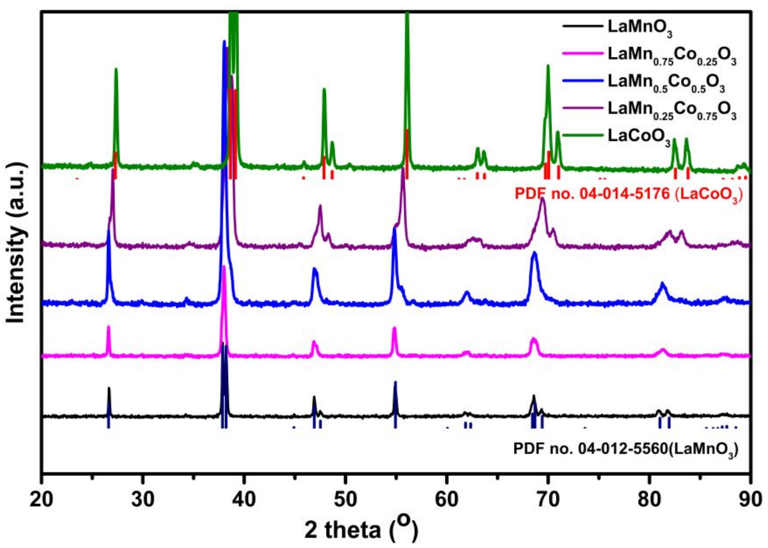


Figure S2. The XRD diffraction of Co-doped $LaMnO_3$ nanoparticles.

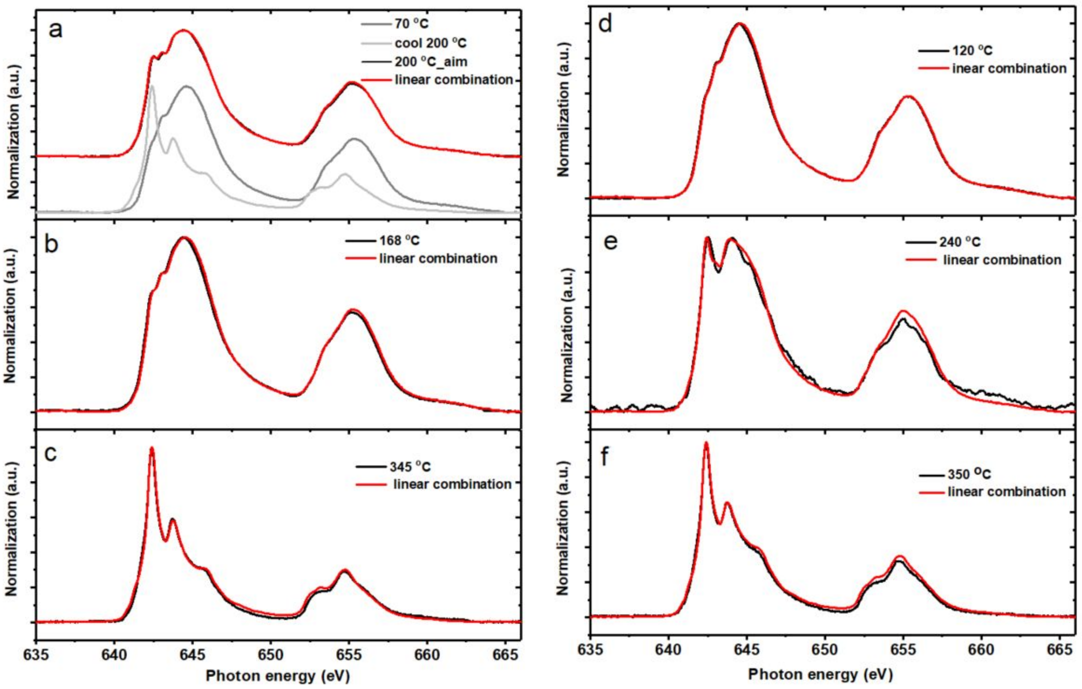


Figure S3. (a-f) The linear combination fitting of the Mn 2p XAS spectra in the thermal study of the $LaMnO_3$ sample: using 70 $^{o}$C Mn 2p XAS spectra (equal to r.t. Mn 2p XAS spectra, i.e. $Mn^{3+}$) and cool 200 $^{o}$C Mn 2p XAS spectra (i.e. $Mn^{2+}$) taking from Figure 1 as references to match aim spectra of 200 $^{o}$C, 120 $^{o}$C, 168 $^{o}$C, 240 $^{o}$C, 345 $^{o}$C, and 350 $^{o}$C Mn 2p XAS to figure out the weights of $Mn^{2+}$ and $Mn^{3+}$.

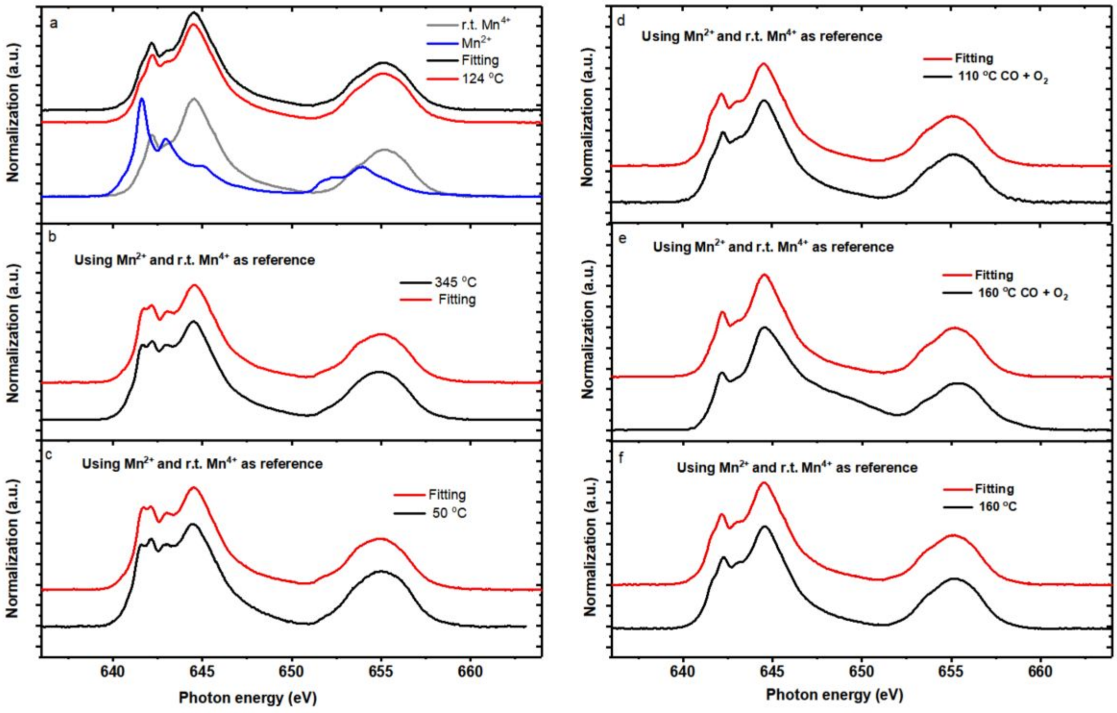

Figure S4. (a-f) The linear combination fitting of the Mn 2p XAS spectra of the $LaMn_{0.5}Co_{0.5}O_3$ sample under operando conditions: using $Mn^{2+}$ 2p XAS spectra ($Mn^{2+}$ in Figure 1) and r.t. $Mn^{4+}$ spectra (i.e. room temperature Mn 2p XAS spectra of $LaMn_{0.5}Co_{0.5}O_3$) as references to match the spectra of 124 °C, 345 °C, 50 °C, 110 °C CO+$O_2$ and 160 °C CO+$O_2$, in order to estimate the average oxidation state.

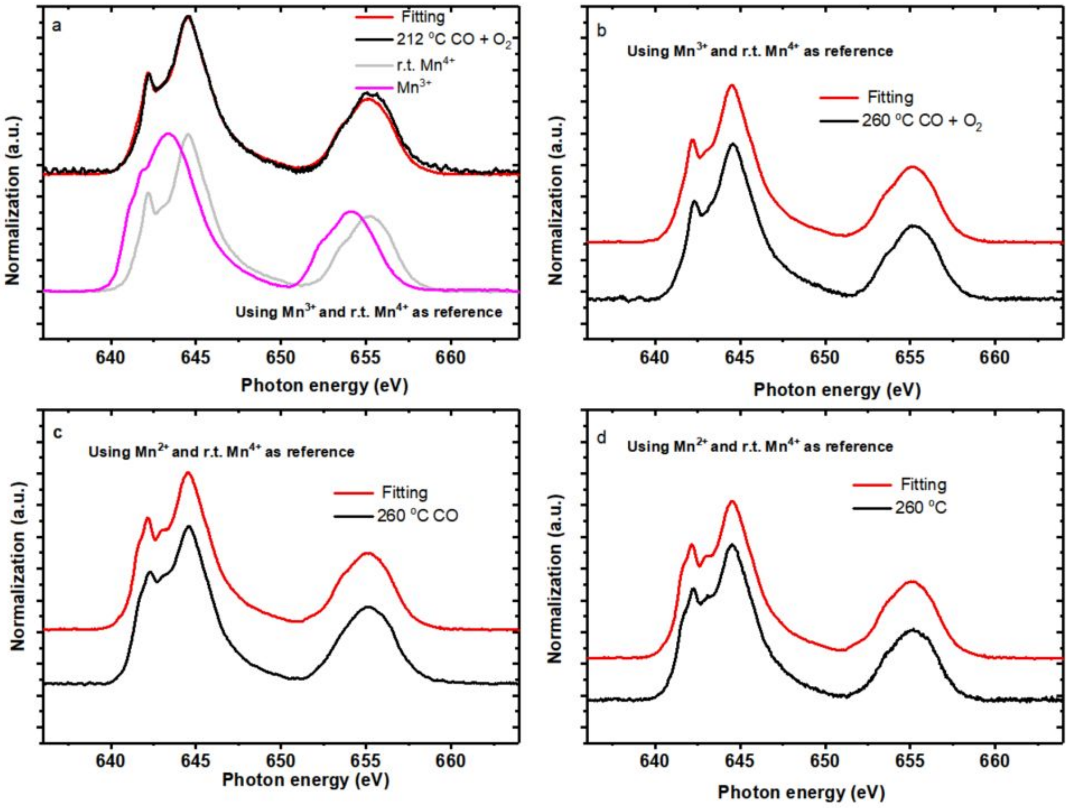


Figure S5. (a-d) The linear combination fitting of the Mn 2p XAS spectra of the $LaMn_{0.5}Co_{0.5}O_3$ sample under operando conditions: using Figure 1 Mn2p XAS spectra ($Mn^{2+}$ and $Mn^{3+}$) and r.t. $Mn^{4+}$ spectra (i.e. room temperature Mn 2p XAS spectra of $LaMn_{0.5}Co_{0.5}O_3$) as references to match aim spectra of 212 °C CO+$O_2$, 260 °C CO+$O_2$, 260 °C CO, and 260 °C Mn 2p XAS to estimate the average oxidation state.

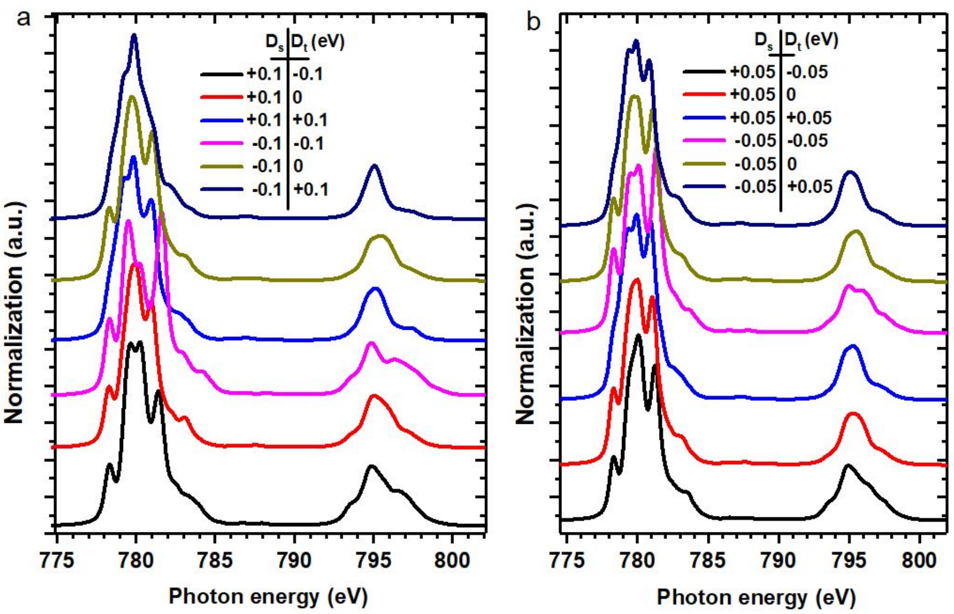


Figure S6. (a,b) The $Co^{2+}$ 2p XAS simulations in $D_{4h}$ symmetry with different Ds and Dt values as indicated in the insets; all other parameters are kept constant.

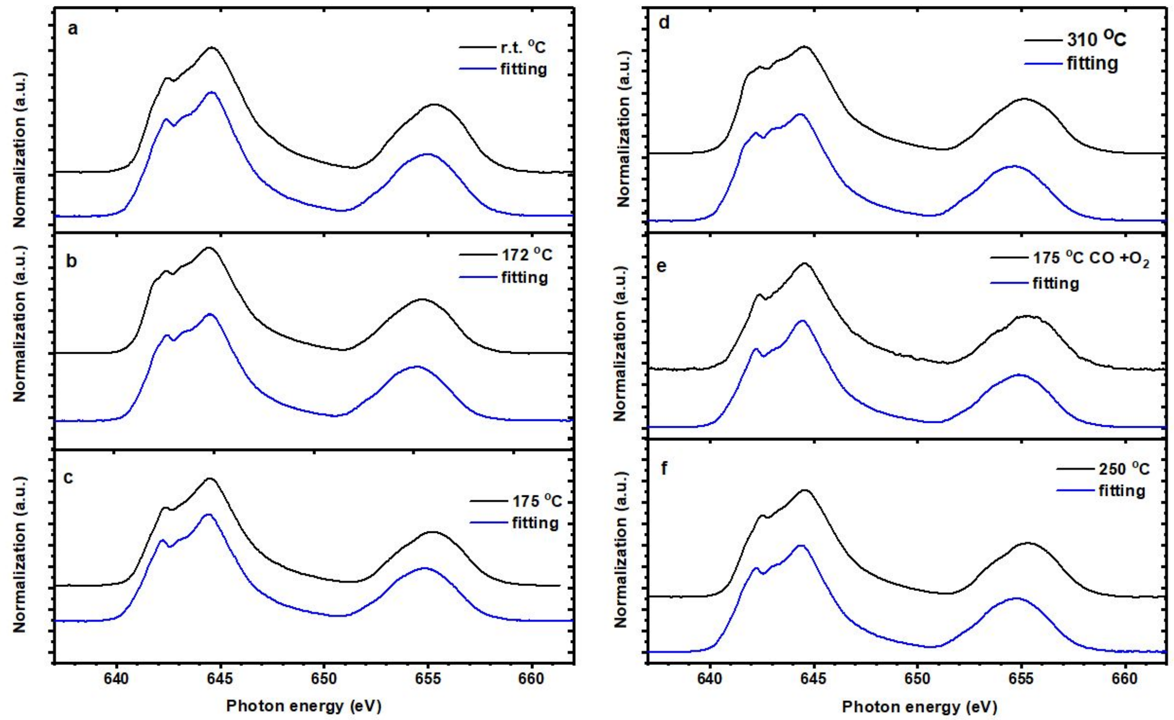


Figure S7. (a-f) The linear combination fitting of the Mn 2p XAS spectra of the $LaMn_{0.75}Co_{0.25}O_3$ sample under operando conditions: using Figure S1 Mn2p XAS spectra ($Mn^{2+}$ and $Mn^{3+}$) and r.t. $Mn^{4+}$ spectra (i.e. room temperature Mn 2p XAS spectra of $LaMn_{0.5}Co_{0.5}O_3$) as references to match the spectra of r.t $^{o}C$, 172 $^{o}C$, 310 $^{o}C$, 175 $^{o}C$ CO+$O_2$, 175 $^{o}C$ and 250 $^{o}C$ in order to estimate the population of $Mn^{2+}$, $Mn^{3+}$, $Mn^{4+}$ and the average oxidation state.

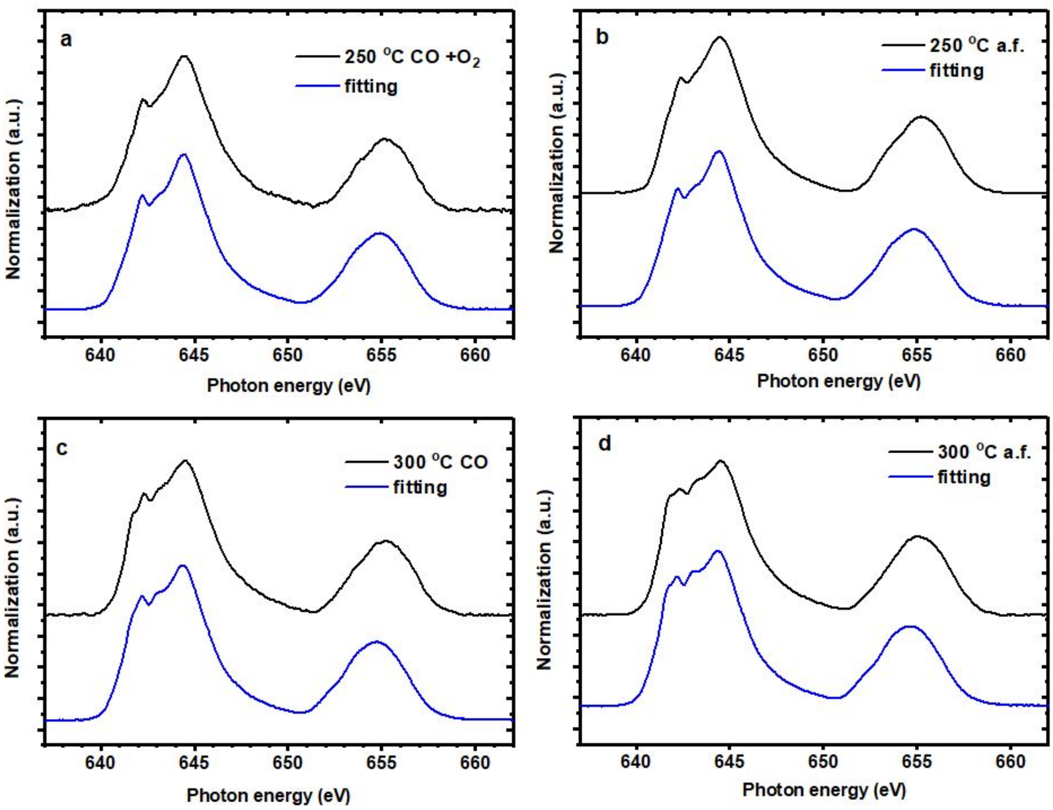


Figure S8. (a-d) The linear combination fitting of the Mn 2p XAS spectra of the $LaMn_{0.75}Co_{0.25}O_3$ sample under operando conditions: using above Figure S1 Mn2p XAS spectra ($Mn^{2+}$ and $Mn^{3+}$) and r.t. $Mn^{4+}$ spectra (i.e. room temperature Mn 2p XAS spectra of $LaMn_{0.5}Co_{0.5}O_3$) as references to match the spectra of 250 °C CO+$O_2$, 250 °C a.f., 300 °C CO, 300 °C a.f., in order to estimate the population of $Mn^{2+}$, $Mn^{3+}$, $Mn^{4+}$ and the average oxidation state.

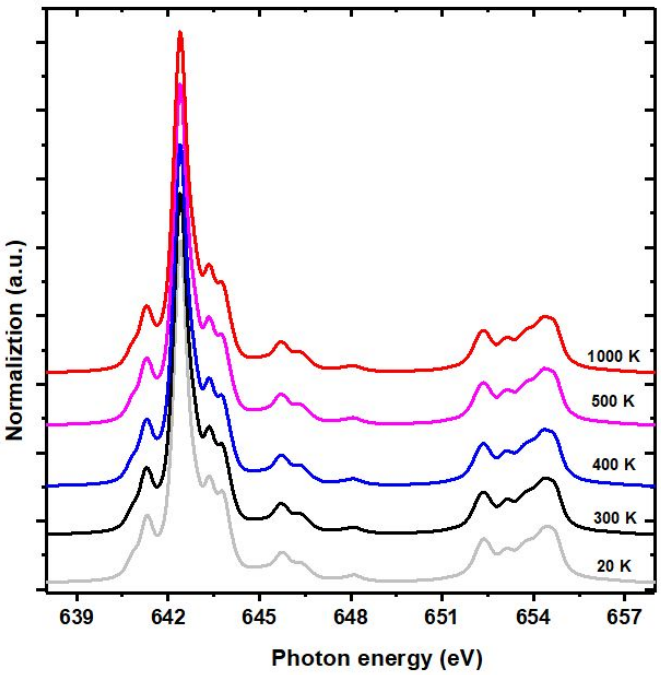


Figure S9. (a) The Mn 2p XAS simulations of MnO by charge transfer multiplet theory from 20 K to 1000 K (The parameters see Table S2), in which the spectra is not sensitive by temperature.

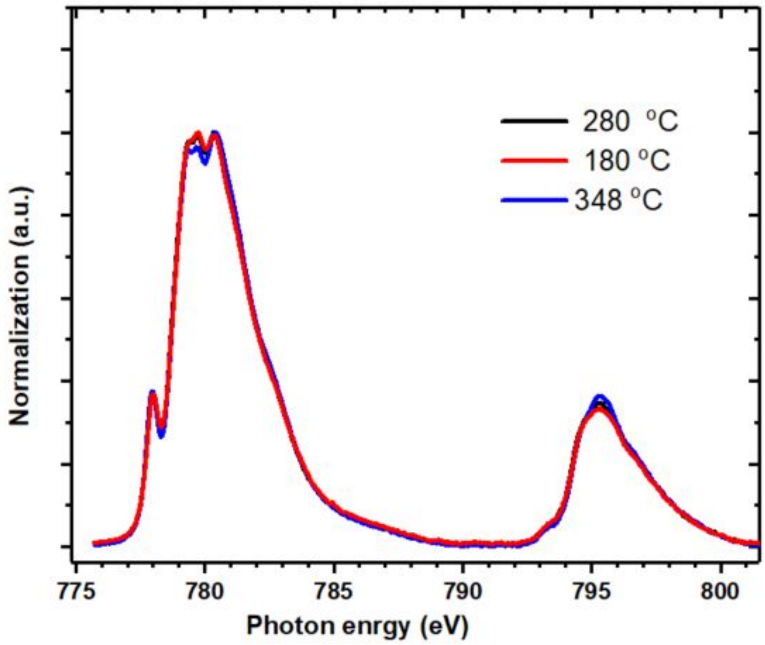


Figure S10 . The Co 2p XAS from $LaCo_{0.5}Mn_{0.5}O_3$ sample at 348 °C, 280 °C, and 180 °C used as reference spectra for the linear combination fitting of the operando $LaCoO_3$ spectra. The minor temperature dependence is due to the spin-orbit split ground state of $Co^{2+}$.

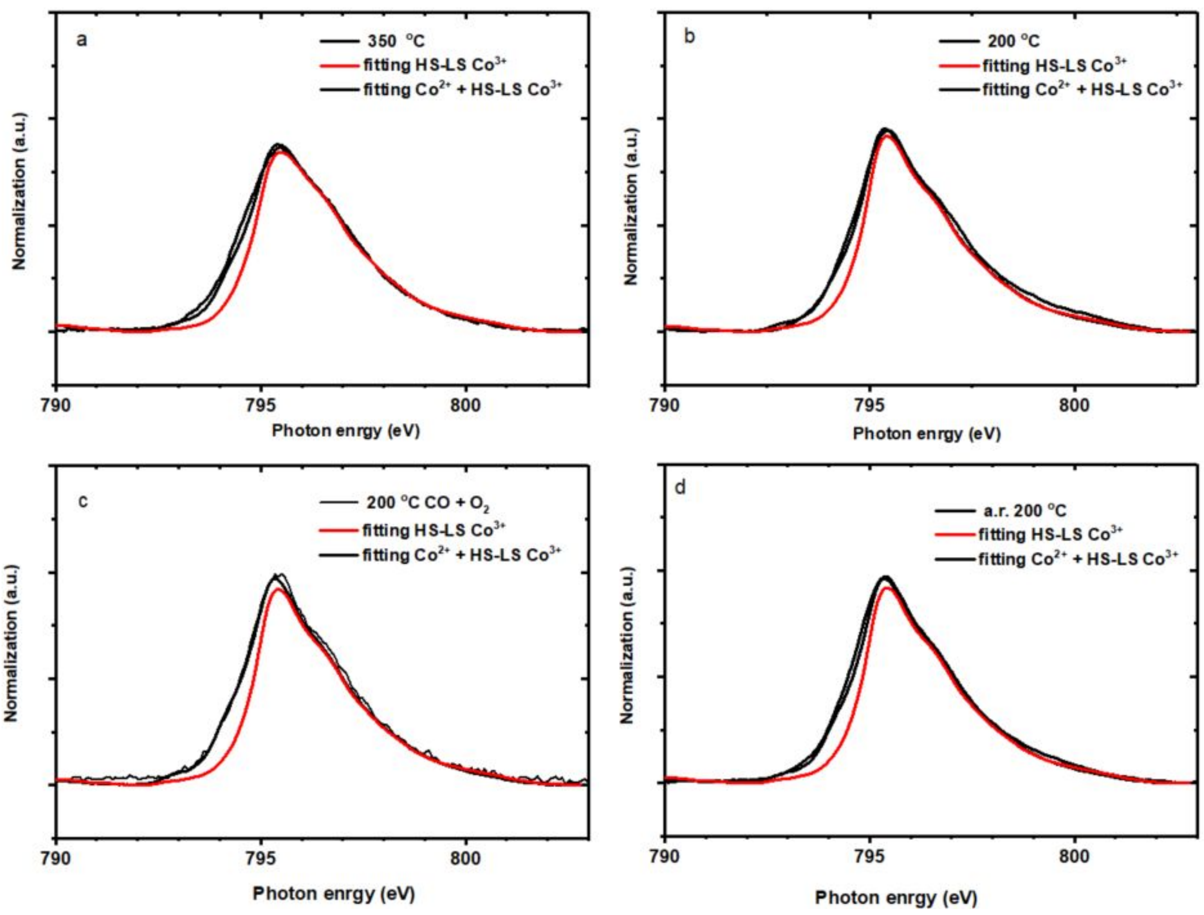


Figure S11. (a-d) The linear combination fitting Co 2p XAS spectra of operando $LaCoO_3$ sample at the condition of 350 °C, 200 °C, 200 °C CO +$O_2$ , a.r. 200 °C (a.r.: after reaction) using reference $Co^{2+}$, $Co^{3+}$ HS- LS from Figure S8 and Figure S7, respectively.

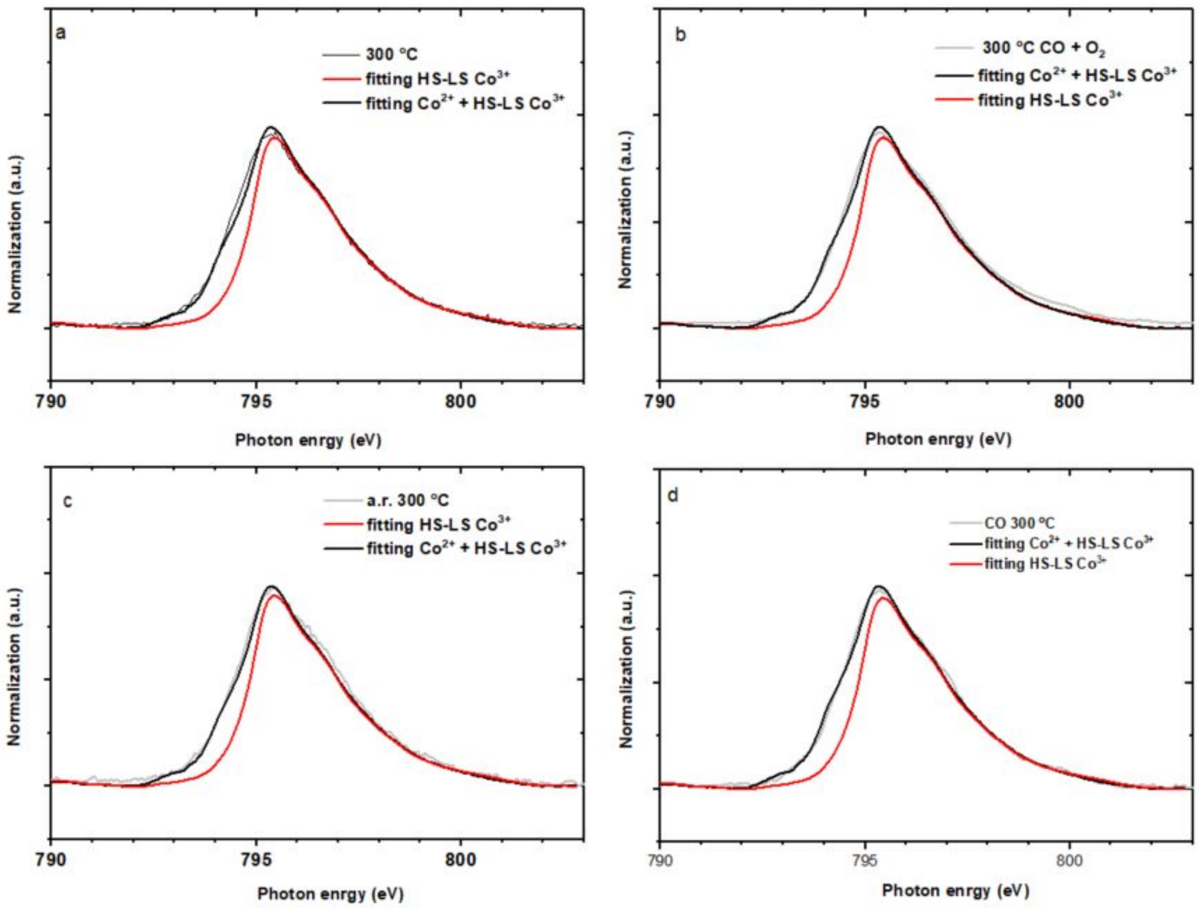


Figure S12. (a-d) The linear combination fitting Co 2p XAS spectra of operando $LaCoO_3$ sample at the condition of 300 ºC, 300 ºC CO + $O_2$, a.r. 300 ºC , CO 300 ºC using references for $Co^{2+}$ and $Co^{3+}$ (HS and LS) from Figure S8 and Figure S7, respectively.

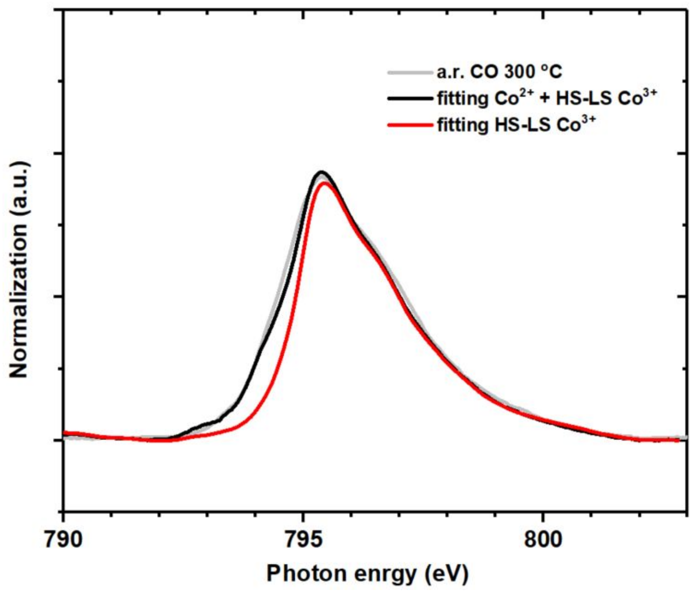


Figure S13. The linear combination fitting Co 2p XAS spectra of operando $LaCoO_3$ sample at a.r. CO 300 $^{o}C$ using reference $Co^{2+}$, $Co^{3+}$ HS- LS from Figure S8 and Figure S7, respectively.

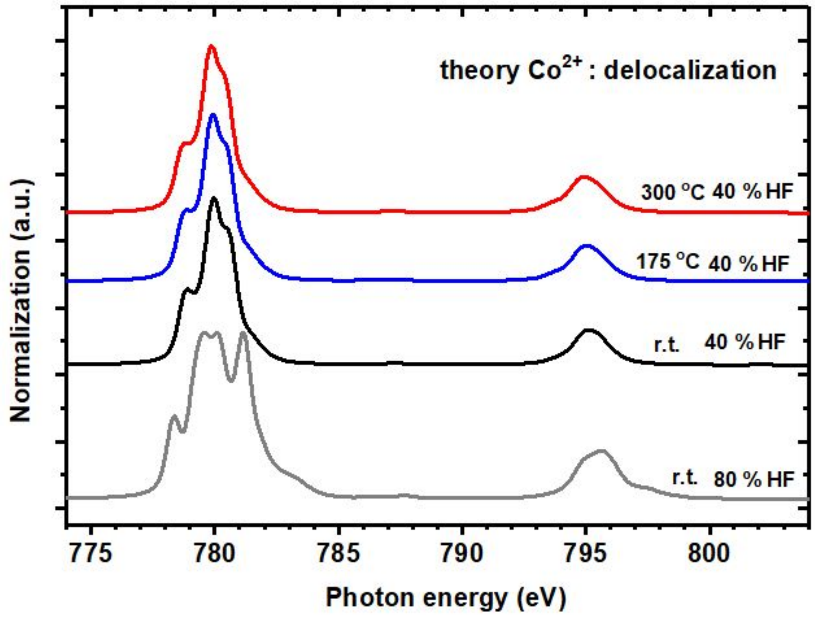


Figure S14. The HF values and temperature effects in Oh $Co^{2+}$ for delocalization.

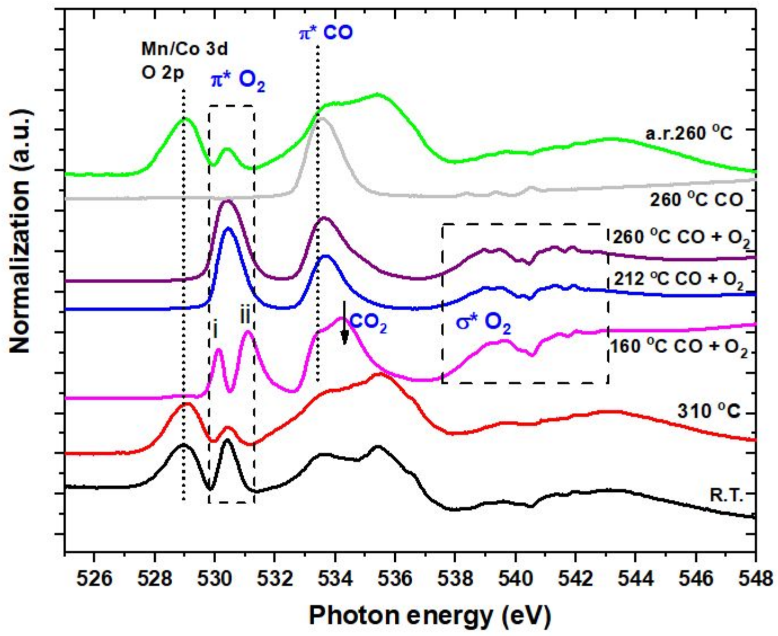


Figure S15. The operando O 1s XAS of $LaMn_{0.5}Co_{0.5}O_3$ in the CO oxidation reaction process at 1 bar pressure. At 160 °C CO + $O_2$ condition, i and ii indicates two possible oxygen-intermediate species.

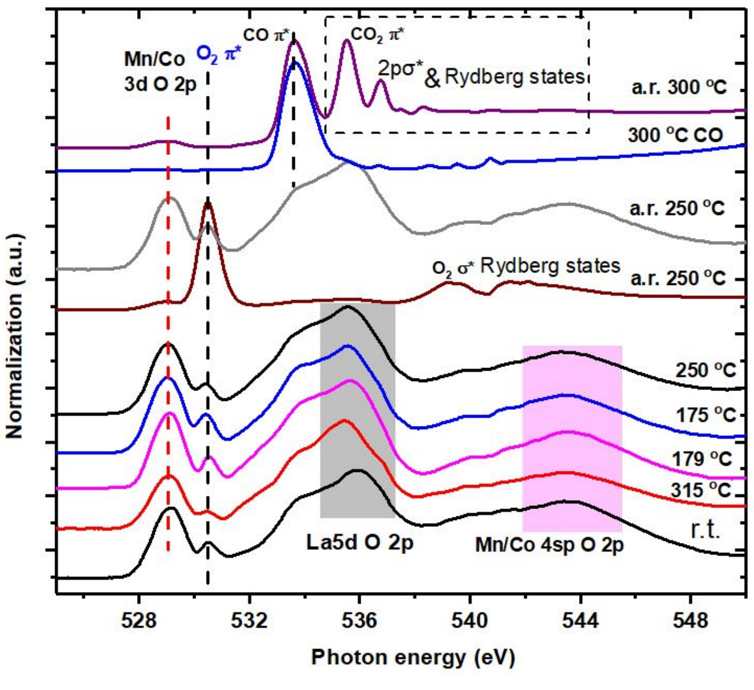


Figure S16. The operando O 1s XAS of $LaMn_{0.75}Co_{0.25}O_3$ in the CO oxidation reaction process at 1 bar pressure.

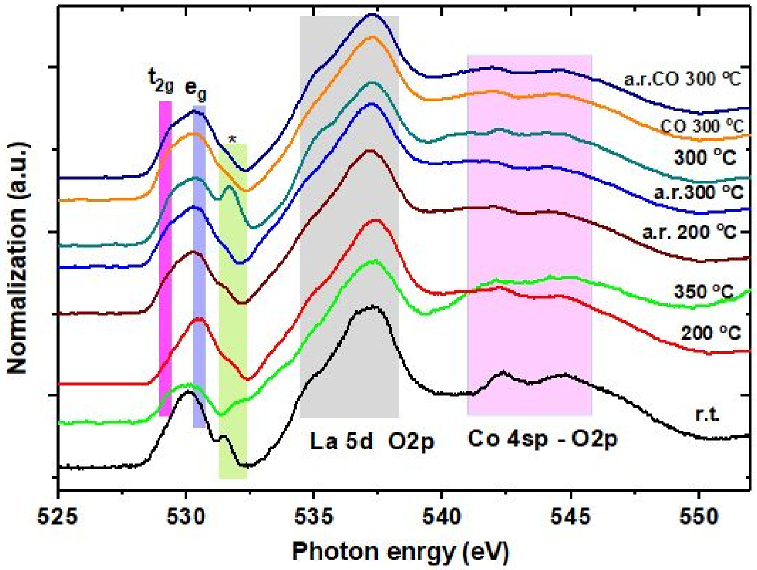


Figure S17. The operando O 1s XAS of $LaCoO_3$ in the CO oxidation reaction process at 1 bar pressure. The '*' indicates $O_2$ plus absence or presence of $Co^{2+}$.

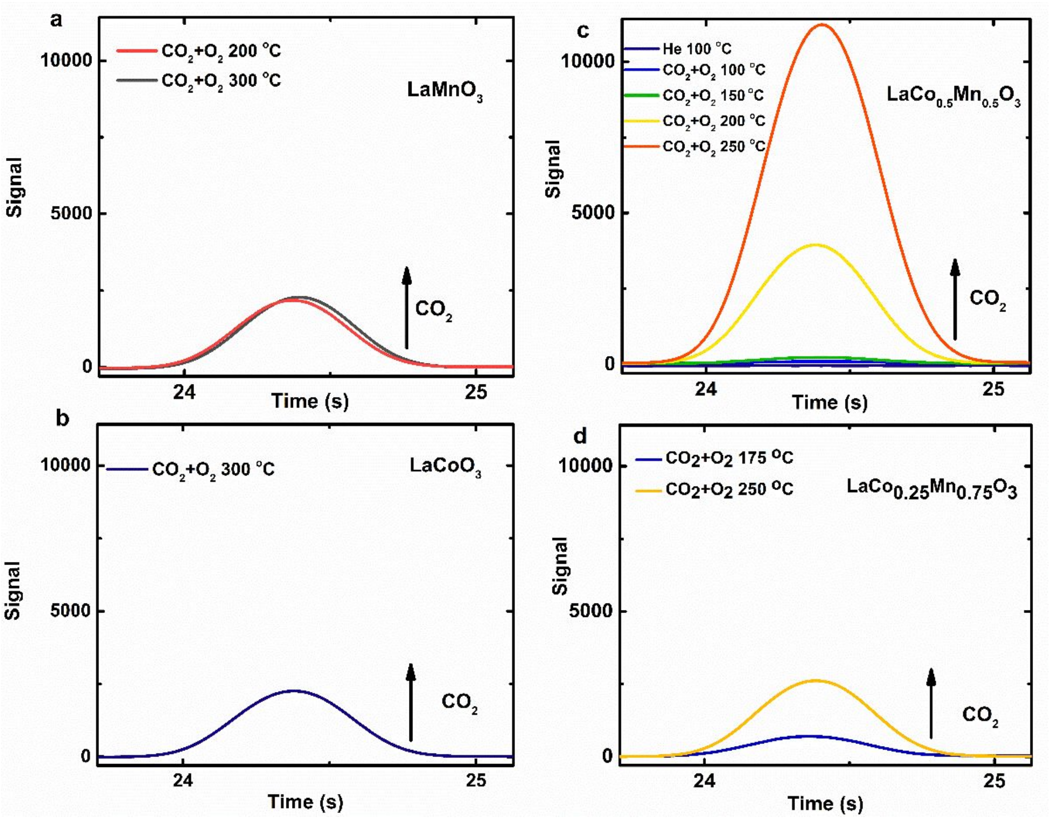


Figure S18. The $CO_2$ products measured in Micro Gas Chromatography (GC) during operando XAS.

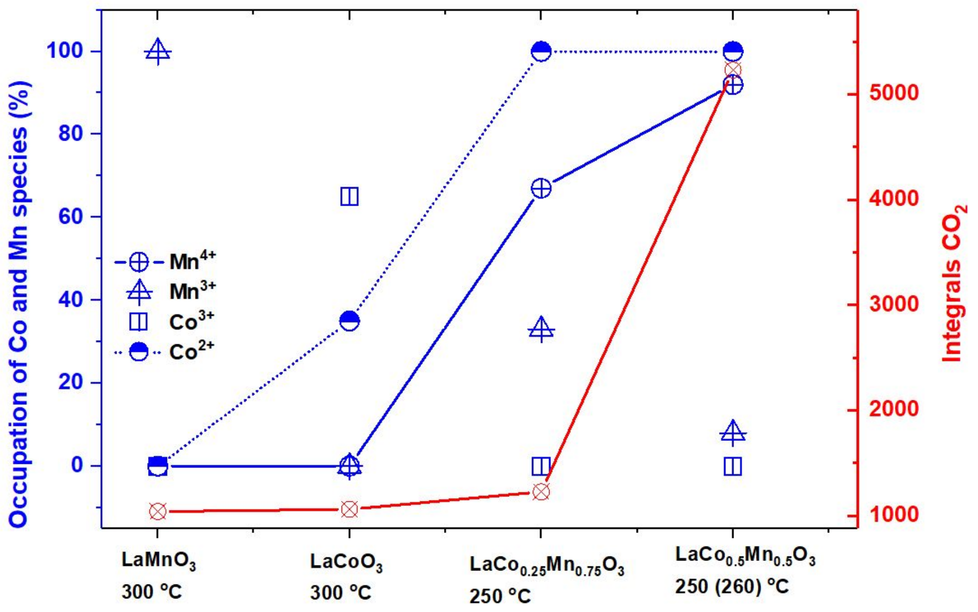


Figure S19. The components Co-Mn oxidation states and activity (integrals of $CO_2$ products from Figure S18) of four catalysts during operando CO + $O_2$.

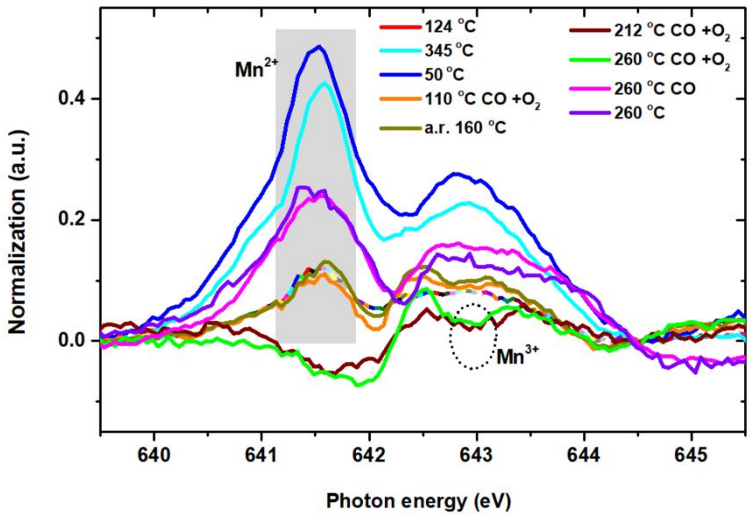


Figure S20. The Mn 2p XAS difference spectra of $LaCo_{0.5}Mn_{0.5}O_3$ using R.T. spectra as reference from Figure 3c.

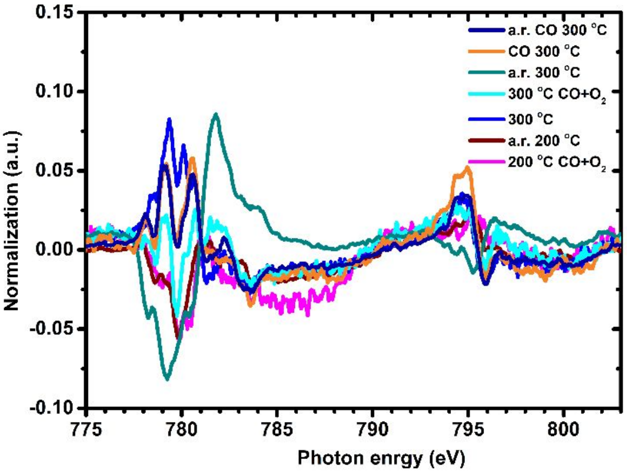


Figure S21. The Mn 2p XAS difference spectra of $LaMn_{0.75}Co_{0.25}O_3$ using R.T. spectra as reference from Figure 5a.

Table S1 The grain sizes of $LaMn_{1-x}Co_xO_3$ (x=0, 0.25, 0.5, 0.75, 1) nanoparticles

| Nanoparticles | average sizes (nm) | wavelength | XRD source |
|---|---|---|---|
| $LaMnO_3$ | 35.66 | 1.78897Å | Co Kα1 |
| $LaMn_{0.75}Co_{0.25}O_3$ | 26.76 | | |
| $LaMn_{0.5}Co_{0.5}O_3$ | 19.30 | | |
| $LaMn_{0.25}Co_{0.75}O_3$ | 20.50 | | |
| $LaCoO_3$ | 34.62 | | |

Table S2 parameters of $MnO_6$ clusters in charge transfer multiplet simulations (eV)

| Cluster | $\Delta$ | $U_{dd}$ | $U_{pd}$ | $V_{eg}$ | $V_{t2g}$ | [a]HF value | 10Dq | $\zeta_{2p}$ | $\zeta_{3d}$ | T (K) |
|---|---|---|---|---|---|---|---|---|---|---|
| $MnO_6$ (+2) | 8.0 | 5.5 | 7.2 | 1.92 | 1.15 | 80 % | $O_h$ (0.56) | 6.85 | 0.040 | 20-1000 |
| $MnO_6$ (+3) | 3 | 7.5 | 9.04 | $V_{a1g}$=2.545<br>$V_{b1g}$=4.408<br>$V_{b2g}$=2.204<br>$V_{eg}$=3.118 | | 80% | $D_{4h}$ (2)<br>$\Delta s$=0.2<br>$\Delta t$=0.05 | 6.85 | 0.046 | 300-1000 |
| $MnO_6$ (+4) | -3 | 5 | 6 | 2.77 | 1.48 | 70 % | [b]0.95-2.31 | 6.86 | 0.052 | 300 |

[a] The reduction of the Hartree-Fock values for $F^2_{dd}$ , $F^4_{dd}$, $F^2_{pd}$, $G^1_{pd}$, and $G^3_{pd}$.

[b]The optimized crystal splitting value of 1.7 eV for $Mn^{4+}$.

Table S3 parameters of $CoO_6$ clusters in charge transfer multiplet simulations (eV)

| $CoO_6$ | Symmetry | $U_{dd}$ | $U_{pd}$ | $\Delta$ | 10Dq | $V_{t2g}$ | $V_{eg}$ | $\zeta_{3d}$ | T (K) |
|---|---|---|---|---|---|---|---|---|---|
| HS ($Co^{2+}$) | $O_h$ | 6.5 | 8.2 | 6.5 | 0.75 | 1.8 | | 0.066 | 0- |
| HS ($Co^{2+}$) | $D_{4h}$ | 6.5 | 8.2 | 6.5 | 0.75 | 1.05<br>$V_{b1}$= 1.8<br>$V_{a1}$ = 1.8<br>$V_{b2}$ = 1.05<br>Ve= 1.05 | | or 0 | 1000 |

Slater-integrals are reduced to 80% of Hartree-Fock values for $F^2_{dd}$ , $F^4_{dd}$, $F^2_{pd}$, $G^1_{pd}$, and $G^3_{pd.}$

References


(1) Fracchia, M.; Ghigna, P.; Pozzi, T.; Anselmi Tamburini, U.; Colombo, V.; Braglia, L.; Torelli, P. Stabilization by Configurational Entropy of the Cu(II) Active Site during CO Oxidation on $Mg_{0.2}Co_{0.2}Ni_{0.2}Cu_{0.2}Zn_{0.2}O$. *J. Phys. Chem. Lett.* 2020, 11, 9, 3589–3593.

(2) Niwa, E.; Uematsu, C.; Miyashita, E.; Ohzeki, T.; Hashimoto, T. Conductivity and Sintering Property of $LaNi_{1-x}Fe_xO_3$ Ceramics Prepared by Pechini Method. *Solid State Ion.* **2011**, *201* (1), 87–93.
